# Development of a Fingerprint-Based Scoring Function for the Prediction of the Binding Mode of Carbonic Anhydrase II Inhibitors

**DOI:** 10.3390/ijms19071851

**Published:** 2018-06-23

**Authors:** Giulio Poli, Vibhu Jha, Adriano Martinelli, Claudiu T. Supuran, Tiziano Tuccinardi

**Affiliations:** 1 Department of Pharmacy, University of Pisa, 56126 Pisa, Italy; giulio.poli@unipi.it (G.P.); vibhujha16@gmail.com (V.J.); adriano.martinelli@unipi.it (A.M.); 2 NEUROFARBA Department, Sezione di Scienze Farmaceutiche e Nutraceutiche, Università degli Studi di Firenze, Sesto Fiorentino, 50019 Florence, Italy; claudiu.supuran@unifi.it

**Keywords:** carbonic anhydrase II inhibitor, docking, scoring function

## Abstract

Carbonic anhydrase II (CAII) is a zinc-containing metalloenzyme whose aberrant activity is associated with various diseases such as glaucoma, osteoporosis, and different types of tumors; therefore, the development of CAII inhibitors, which can represent promising therapeutic agents for the treatment of these pathologies, is a current topic in medicinal chemistry. Molecular docking is a commonly used tool in structure-based drug design of enzyme inhibitors. However, there is still a need for improving docking reliability, especially in terms of scoring functions, since the complex pattern of energetic contributions driving ligand–protein binding cannot be properly described by mathematical functions only including approximated energetic terms. Here we report a novel CAII-specific fingerprint-based (IFP) scoring function developed according to the ligand–protein interactions detected in the CAII-inhibitor co-crystal structures of the most potent CAII ligands. Our IFP scoring function outperformed the ability of Autodock4 scoring function to identify native-like docking poses of CAII inhibitors and thus allowed a considerable improvement of docking reliability. Moreover, the ligand–protein interaction fingerprints showed a useful application in the binding mode analysis of structurally diverse CAII ligands.

## 1. Introduction

Carbonic anhydrases (CAs) belong to the superfamily of ubiquitous metalloenzymes and are characterized by the presence of a zinc metal ion in their active sites [1]. They are present in both prokaryotes and eukaryotes, with a major role in catalyzing the reversible hydration of carbon dioxide to carbonic acid [2]. Seven genetically distinct CA families (α, β, γ, δ, ζ, η, θ) have been discovered and 16 human α-CA isozymes differing in their catalytic activity, distribution, and cellular localization have been identified [3]. Among these isoforms, some are cytosolic (CAI, CAII, CAIII, CAVII, and CAXIII), others are membrane-bound isozymes (CAIV, CAIX, CAXII, CAXIV, and CAXV), two of them are mitochondrial (CAVA and CAVB), and one isozyme is secreted in saliva (CAVI). These isozymes are critically involved in various physiological processes such as pH regulation, respiration, electrolyte secretion, bone reabsorption, calcification, gluconeogenesis, neurotransmission, lipogenesis, and ureagenesis across different tissues and organs [4,5]. Due to the vast range of physiological roles played by CAs, an aberrant activity of most of CA isoforms is associated with different diseases such as glaucoma, epilepsy, osteoporosis, obesity, and various types of tumors [6]. Therefore, CA inhibitors are considered as promising therapeutic agents for the treatment of these pathologies [7]. Among the various CA isoforms, carbonic anhydrase II (CAII) was found be to the most active one and it is abundantly expressed in most organs. In particular, it is considered as an extremely important target for diuretics, anti-osteoporosis, anti-glaucoma, and especially anti-tumor drugs [8,9]. Due to its active participation in different physiological processes and its involvement in various diseases, CAII represents a widely studied target for drug design and the identification of new CAII inhibitors is still a hot topic in medicinal chemistry. Predominantly sulfonamides and coumarins have been used to inhibit the catalytic activity of CAs with different mechanisms of enzyme inhibition. Sulfonamides coordinate the cofactor Zn^2+^ ion in the active sites of CAs thus inhibiting their catalytic activity, whereas coumarins have shown a completely different mode of inhibition by binding to the entrance of CAs’ active site without interacting with the Zn^2+^ ion [7]. In the present study, we have investigated the possibility of developing a docking protocol calibrated for the prediction of the binding disposition of CAII inhibitors. The identification of the actual binding mode of a ligand into a target receptor through molecular docking is still a major challenge in the field of structure-based drug design [10]. A docking algorithm is basically composed by two different elements addressing the two main issues connected to the prediction of a putative ligand binding mode. The first element is a search engine (often a Monte Carlo or genetic algorithm) exploring the conformational space of the ligand and identifying the possible low-energy dispositions that the molecule can assume within the protein binding site. The second element is a scoring function that is employed to calculate the ligand–protein binding energy associated to the different docking poses generated by the search engine. Therefore, the scoring function performs the task of evaluating the reliability of the docking poses from an energetic point of view and selecting the most energetically favored one, which should represent the binding mode that is most likely to be assumed by the ligand. Several different empirical, knowledge-based and force field-based scoring functions have been implemented in the plethora of docking software currently available. However, very often the standard scoring functions are unable to discriminate the native-like docking poses from a large set of generated ligand dispositions, because protein–ligand binding is driven by a complex pattern of energetic contributions that cannot be fully described by mathematical functions that only include approximated energetic terms. In this context, the use of protein–ligand interaction fingerprints has been recently introduced as a promising tool in structure-based drug design and particularly in docking studies, because it outperforms conventional scoring functions and improves the identification of accurate ligand binding modes, thus facilitating the discovery of active compounds through virtual screening studies [11]. The fingerprint-based post-docking procedure involves encoding significant protein–ligand interactions detected from co-crystal structures into fingerprints. The generated interaction fingerprints allow a better assessment of ligand docking poses based on their similarities to the crystallographic binding modes of the reference compounds. Such similarity is then quantified in the form of a binding score that is used to prioritize the most reliable docking poses among the set of docking solutions produced for a single ligand. Moreover, fingerprints can also be used to analyze and compare target protein complexes including structurally diverse ligands [12]. Therefore, in order to rely on an efficient post-docking processing for the analysis of CAII inhibitors, we herewith propose the use of ligand–protein interaction fingerprints (IFPs) [13] for prioritizing the most relevant poses among the ones generated by docking calculations.

## 2. Results and Discussion

**Figure 1:**
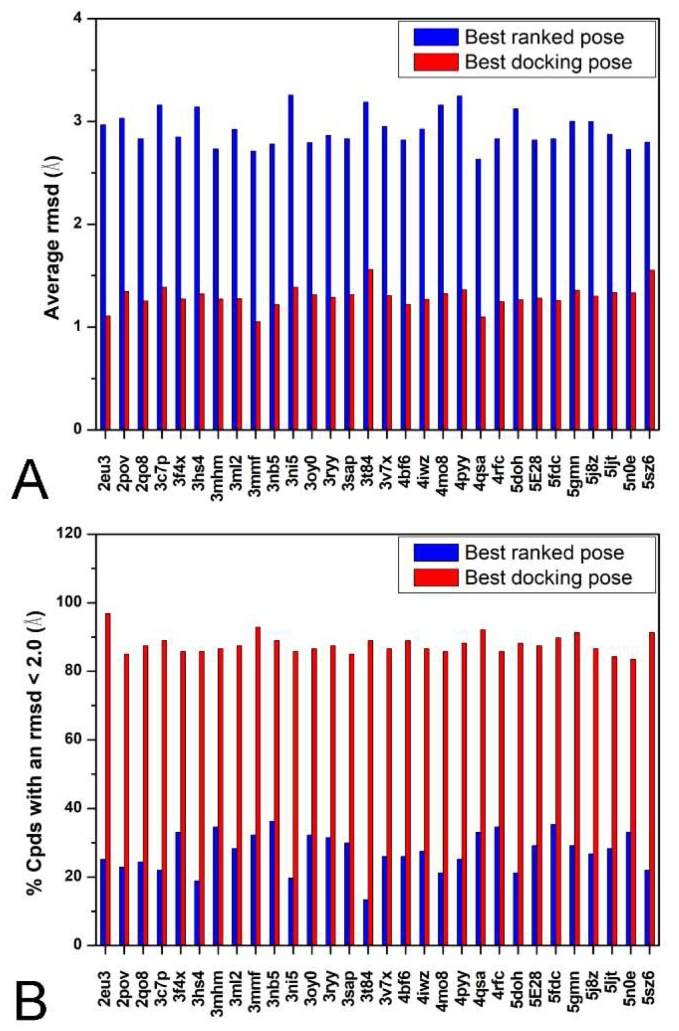
Results of the cross-docking study. For each carbonic anhydrase II (CAII) protein, the average root-mean-square deviation (aRMSD) (**A**) and the percentage of poses with a RMSD less than 2.0 Å (**B**) are reported for both the best ranked pose (blue) and the best binding disposition among the 100 generated docking poses (red).

As a first step, cross-docking studies were performed to evaluate the reliability of Autodock4 software for predicting the binding disposition of CAII inhibitors within the catalytic site of the enzyme. As reported in the Materials and Methods Section 3, a total of 127 CAII-inhibitor X-ray complexes were downloaded from the Protein Data Bank (PDB) (see Table S1) [14]; all co-crystallized ligands were then extracted from their corresponding X-ray complex and subjected to conformational search. Subsequently, 30 CAII crystal structures were randomly selected among the downloaded complexes and the 127 ligands were docked into these 30 CAII protein structures. By applying this procedure, 3810 docking results (top-scored solutions) were thus obtained. Then, the reliability of Autodock4 software was evaluated by comparing the docking poses generated for each ligand with the corresponding crystallographic pose (see Materials and Methods Section 3.3 for details). In particular, we calculated the average root-mean-square deviation (aRMSD) of the ligands’ docking poses with respect to their experimental dispositions. On these bases, the aRMSD corresponded to the mean RMSD value obtained from all the ligands docked into the 30 CAII structures. Additionally, the number of ligands with a reliable docking pose (NLRD), presenting an RMSD below 2.0 Å, was also calculated and used for the statistical analysis [15,16]. As shown in Figure 1, Autodock4 performed quite well as it showed an aRMSD of 2.9 Å and a NLRD of 27.4%. At this point, we carried out a similar analysis where, for each ligand docked into each of the 30 CAII binding sites, 100 different docking results were considered, instead of just the top-scored docking pose, and the RMSD of the docking solution with the closest coordinates to the experimental pose (native-like pose) was used for the statistical analysis. In this way, we obtained an aRMSD of 1.3 Å and a NLRD of 87.9%, indicating that the software was able to correctly provide a highly reliable ligand pose among the 100 generated docking results. Indeed, by measuring the RMSD of such a wide set of docking poses for each ligand, we virtually excluded the contribution of the scoring step to the docking process and we were thus able to verify the ability of the Autodock4 search engine to identify a reliable native-like pose independently from the rescoring evaluation. Therefore, these results could be also considered as the best results obtainable through this software if an ideal, optimal scoring function was used for ranking the generated solutions. In Figure 1, the quality of Autodock4 top-scored poses is compared to the quality of the native-like poses generated by its search algorithm. In summary, with the first step of our analysis we can conclude that Autodock4 algorithm performs quite well in the prediction of CAII-inhibitor interactions but the associated scoring function could be further improved, since the search algorithm is able to generate native-like docking poses that the scoring function is unable to rank as the top-scored ones.

In order to develop a scoring function specific for the CAII-inhibitor binding prediction, a fingerprint approach, able to describe the ligand–protein interactions pattern was employed. For this purpose, the interactions of the 127 co-crystalized inhibitors with CAII have been systematically evaluated by using BINANA 1.2.0 [17]. This software is able to automatically analyze an input ligand–protein complex and detect all the interactions formed by the ligand with the receptor; in particular, the software is able to consider seven different interaction types: H-bond acceptor, H-bond donor, hydrophobic contacts, π—π stacking, T-stacking, cation-π interactions, and salt bridges. Once identified the residues belonging to the CAII binding site, corresponding to 44 residues located in the surrounding of the various co-crystallized ligands (see Figure S4 in the Supporting Information), the interaction of each ligand with each of these residues has been evaluated analyzing the potential presence of the seven interaction types reported above and a binary code has been used for describing them. In case a certain interaction type was present, the value of 1 was associated to that interaction and included in the binary code, whereas a 0 value was reported in case of its absence. In this way, the interaction between each ligand and each binding site residue was described by a binary code composed of seven digits. Table 1 shows a schematic representation of the binary code scheme used for reproducing the interaction of a ligand with one single residue of the binding site. Therefore, by applying this approach, the whole ligand–protein interaction scheme identified in each of the 127 CAII-inhibitor co-crystal structures was described by an interaction fingerprint (IFP) composed of 308 total binary digits (seven potential interactions multiplied by 44 binding site residues). Figure S1 reports, as an example, the fingerprint obtained for the interaction of 5-[(phenylsulfonyl)amino]-1,3,4-thiadiazole-2-sulfonamide into CAII (PDB code 3DBU).

**Figure 2:**
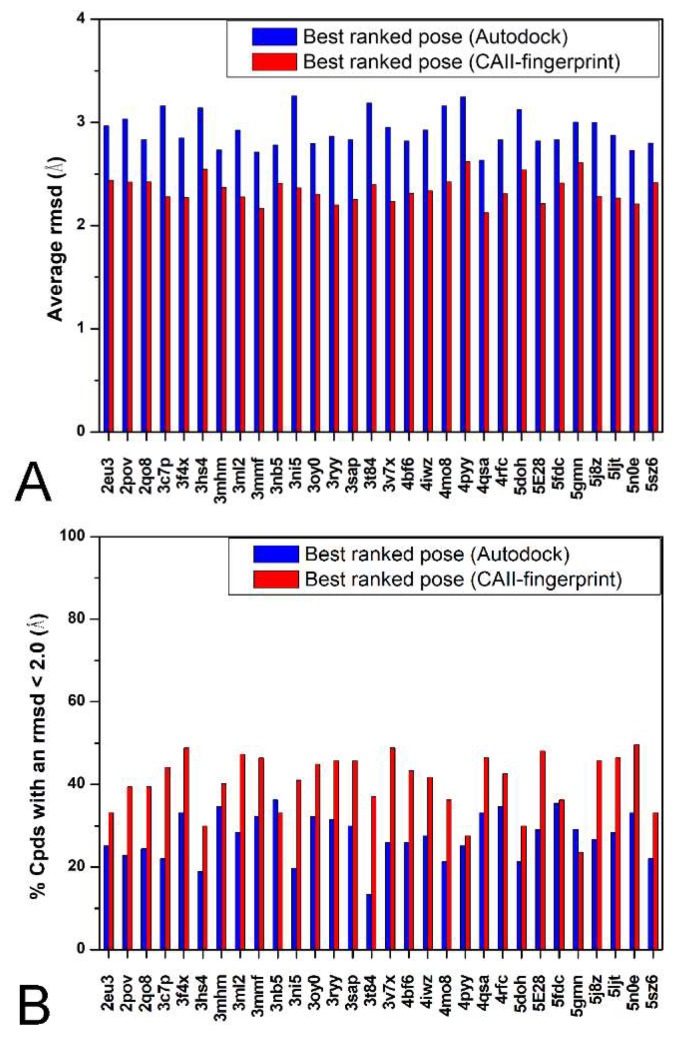
Comparison between the results obtained using the Autodock4 scoring function and the Tanimoto similarity index (Tc-IFP). For each CAII protein, the aRMSD (**A**) and the percentage of poses with a RMSD less than 2.0 Å (**B**) are reported for both the best-ranked pose using the Autodock4 scoring function (blue) and using the Tc-IFP function (red).

As reported in the Materials and Methods Section 3, all the 127 different IFPs were combined into a single reference fingerprint string (termed CAII-rIFP) able to provide a global description of the most relevant interactions formed by the 127 CAII inhibitors inside the enzyme binding site. The CAII-rIFP can be considered as the fingerprint string of an ideal ligand possessing all the possible relevant interactions with the binding site, therefore it was used as a reference for rescoring all the ligands’ poses generated by Autodock4. In more detail, we considered the 100 docking poses previously generated for each of the 127 ligands inside the 30 CAII binding sites and for each docking pose the corresponding fingerprint string was generated and compared with the CAII-rIFP through the calculation of the Tanimoto similarity index (Tc-IFP) value [13] (see the Materials and Methods Section 3). Finally, the Tc-IFP was used as a scoring function for ranking the docking solutions: the docking pose possessing the highest Tc-IFP value among the 100 generated solutions was considered as the best-ranked docking pose and its RMSD value with respect to the corresponding experimental ligand pose was considered for the statistical analysis. Very interestingly, as shown in Figure 2, the application of the Tc-IFP scoring function produced an aRMSD of 2.3 Å and a NLRD of 40.5%, thus resulting in an improvement of the Autodock4 docking reliability compared to that observed when using its own scoring function (aRMSD = 2.9 Å, NRLD = 27.4%) of about 35% for the aRMSD and 13% for NRLD.

**Figure 3:**
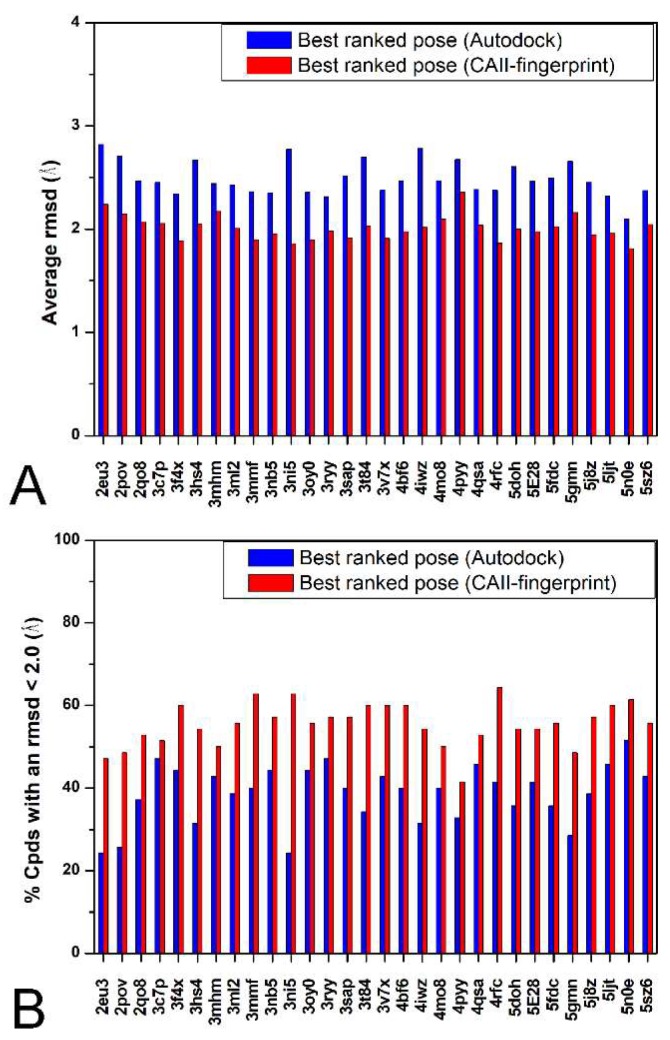
Comparison between the results obtained using the Autodock4 scoring function and the Tc-IFP for the external test set. For each CAII protein, the aRMSD (**A**) and the percentage of poses with a RMSD less than 2.0 Å (**B**) are reported for both the best-ranked pose using the Autodock4 scoring function (blue) and using the Tc-IFP function (red).

The above reported validation has been done rescoring the compounds that were used for developing the CAII-rIFP and could be thus considered as an internal validation of the method. In order to also provide an external validation of the method, X-ray structures of CAII inhibitors that have been reported after the development of our CAII-rIFP or that were not included in the training set because the activity of the co-crystallized ligand was not considered sufficiently high (see Materials and Methods Section 3.1 for details) were downloaded from the Protein Data Bank. As a result, an external test set of 70 CAII inhibitors (see Table S2) was built and then docked into the 30 CAII protein structures previously used. As shown in Figure 3, Autodock4 confirmed its good reliability since, analyzing the best Autodock4 ranked solutions, it showed an aRMSD of 2.5 Å and a NLRD of 38.7%, whereas considering the 100 different docking results generated for each ligand docked into each binding site, we obtained an aRMSD of 0.9 Å and a NLRD of 97.5%. Then, as reported above, the corresponding fingerprint strings were generated for the 100 docking poses of each ligand docked into each protein and compared with the CAII-rIFP through the calculation of the Tc-IFP value. As shown in Figure 3, considering the best docking pose of each ligand as the one with the highest Tc-IFP value, the application of the Tc-IFP scoring function produced an aRMSD of 2.0 Å and a NLRD of 55.5%, thus resulting in an improvement of the Autodock4 docking reliability of about 30% for the aRMSD and 12% for NRLD.

**Figure 4:**
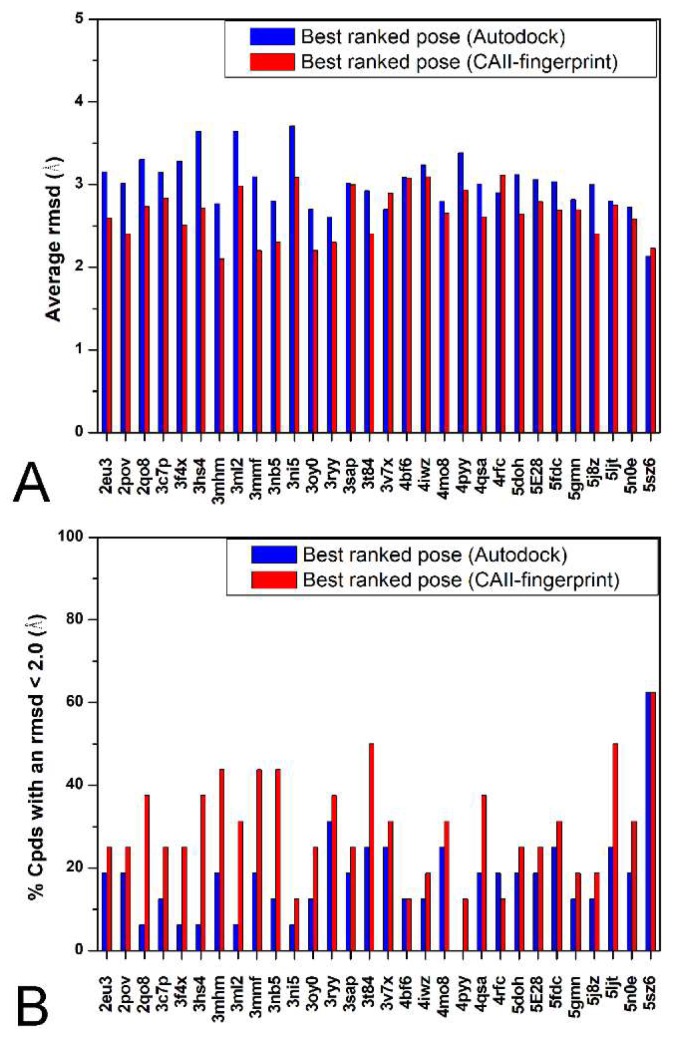
Comparison between the results obtained using the Autodock4 scoring function and the Tc-IFP for the second external test set. For each CAII protein, the aRMSD (**A**) and the percentage of poses with a RMSD less than 2.0 Å (**B**) are reported for both the best-ranked pose using the Autodock4 scoring function (blue) and using the Tc-IFP function (red).

Finally, as all the analyzed ligands contained a sulfonamide moiety as the zinc binding group (ZBG), we built an external test set of 15 CAII inhibitors deposited in the Protein Data Bank that possessed different ZBGs (see Table S3), in order to test if the CAII-rIFP can also be used for compounds with different ZBGs. The docking results for these compounds produced an aRMSD of 3.0 Å and a NLRD of 16.7% considering the best Autodock4 ranked pose (see Figure 4), while the analysis of the RMSD of all the 100 docking poses generated for each ligand docked into each protein resulted in an aRMSD of 1.6 Å and a NLRD of 81.5%. As shown in Figure 4, by applying the CAII-rIFP scoring function we obtained an aRMSD of 2.6 Å and a NLRD of 30.0%, thus resulting in an improvement of about 25% for the aRMSD and 13% for NRLD. Even if these results are a bit worse, in terms of aRMSD, than that obtained in the internal and external validation by using compounds with a sulfonamide moiety, they suggest that the CAII-rIFP could also be profitably used for better predicting the binding mode of CAII inhibitors that do not possess a sulfonamide moiety as the ZBG.

**Figure 5:**
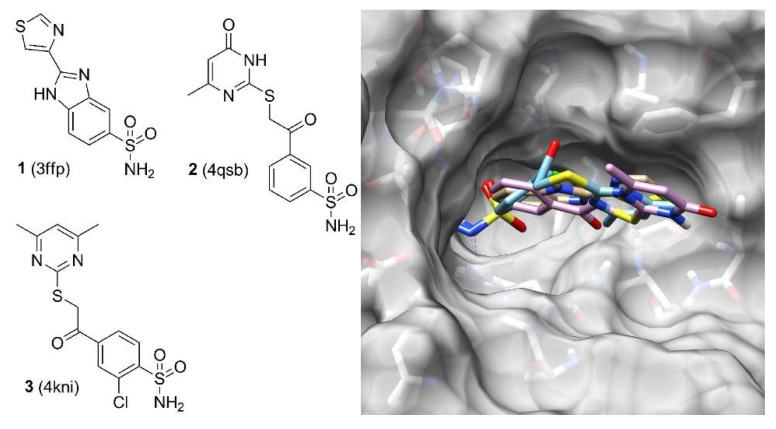
2D structure of compounds **1**, **2,** and **3** and their superimposable disposition into the CAII binding site.

Due to the promising results obtained in terms of docking pose reliability, we also checked the potential use of the fingerprint information for the classification of the CAII inhibitors according to their binding modes. In fact, as reported by de Graaf and co-workers, this kind of approach was already successfully applied for analyzing kinase and phosphodiesterase ligands [18,19]. On these bases, all the compounds showing a similar fingerprint profile (i.e., a Tc-IFP value higher than 0.40) were clustered together (see Materials and Methods Section 3.6 section for details). By applying this method, 110 ligands were grouped in 17 different clusters, whereas the 17 remaining ligands (corresponding to about 13% of the total ligands) showed a unique fingerprint profile (see Figure S2). This clustering approach was able to identify several groups of compounds possessing the same key interactions with the CAII binding site although being characterized by a large diversity in terms of chemical structures. As an example, Figure 5 shows the compounds belonging to the cluster 10 obtained by this analysis: although the structures of these compounds are rather different, they share very similar IFPs and thus common key ligand–protein interactions.

## 3. Materials and Methods

### 3.1. Protein-Ligand Complex Structures

All the CAII-inhibitors co-crystal structures reported in the Protein Data Bank [14] were analyzed and the complexes with CAII inhibitors showing at least an activity in the range of 100 nM were downloaded. As a result, 127 CAII X-ray structures were downloaded and used in this work. Hydrogen atoms, missing residues, and the ligand bond order were added by means of the Protein preparation wizard of Maestro [20], protonation states were chosen by using Propka [21] for the protein residues, and Ionizer [22] for the ligands. Water molecules and ions different by the zinc atom of the binding site were removed. All the protein–ligand complexes were then visually checked. The ligands were then extracted from the X-ray complexes and subjected to a conformational search of 1000 steps in a water environment (using the generalized-Born/surface-area model) by means of the Macromodel software [22]. The algorithm used was the Monte Carlo method with the MMFFs force field and a distance-dependent dielectric constant of 1.0. The same procedure was used for the two external test sets of 70 and 15 CAII inhibitors. The first external test set was constituted by the CAII inhibitors that were deposited after the development of the CAII-rIFP and by the deposited CAII inhibitors that did not show at least an activity in the range of 100 nM (see Table S2). The second external data set was constituted by CAII inhibitors that did not show the sulfonamide group as ZBG (see Table S3).

### 3.2. Docking Procedures

Autodock Tools utilities [23] were used in order to identify the torsion angles in the ligands, to add the solvent model, and assign the Gasteiger atomic charges to proteins and ligands. The regions of interest used by Autodock 4.2 [24] were defined by considering the reference ligand as the central group of a grid box of 10 Å in the *x, y,* and *z* directions. For the Zinc ion, the parameters recently reported by Olson and co-workers were used [25]. A grid spacing of 0.375 Å and a distance dependent function of the dielectric constant were used for the energetic map calculations as reported by Olson and co-workers [25]. By using the Lamarckian genetic algorithm, the docked compounds were subjected to 100 runs of the Autodock search using 2,500,000 steps of energy evaluation and the default values of the other parameters.

### 3.3. Cross-Docking Analysis

The α carbons of the 127 protein structures were aligned with each other using a reference structure. To verify the possible presence of mobile regions that could negatively affect the protein alignment, the secondary structure of the 127 aligned proteins were visualized and, as shown in Figure S3, this analysis highlighted that there were no mobile regions that occupied different positions. Therefore, the alignment of the different protein structures obtained using all the α carbons was considered reliable for the further calculations. Then, in order to reduce the computational effort, 30 out of the 127 selected proteins were randomly chosen and each of the 127 ligands was docked into these 30 CAII structures, thus resulting in a total of 3810 docking calculations. The docking reliability was evaluated by calculating for each ligand and each protein structure the root-mean-square deviation (heavy atoms) between the reference position of the ligand in the experimental CAII-ligand complex and that predicted by the docking software in the various CAII structures [26]. The RMSD analysis was carried out using the rms_analysis software of the GOLD suite [27].

### 3.4. CAII-rIFP Generation

All the residues within the distance of 7 Å of at least one of the 127 ligands were considered as binding site residues, for a total of 44 amino acids (see Figures S1 and S4 in the Supporting Information). The ligand–protein interactions were then analyzed by means of the BINANA software [17]. The hydrogen bond distance and the hydrogen bond angle cutoff were set to 3.5 Å and 50°, respectively, whereas BINANA defaults were used for all other parameters. By using an in-house program, the ligand–protein interactions resulted from the BINANA outputs were converted to binary interaction fingerprint strings (IFPs). Each string was composed by 308 bits, since for each of the 44 residues selected for defining CAII binding site, seven bits indicated the presence (1) or absence (0) of a certain interaction type. The resulting 127 ligand–protein IFPs (one for each ligand-CAII X-ray structure) were then compared to each other and used for generating the CAII-rIFP. Precisely, the CAII-rIFP presented a digit equal to 1 only for the interactions shown by at least three CAII inhibitors. In this way, the interactions shown by too few ligands were considered as noise and excluded from the CAII-rIFP.

### 3.5. Tc-IFP Calculation

A ligand–protein IFP string was generated for each of the 100 docking poses generated for each of the 127 ligands docked into the 30 CAII binding sites by using the method described above. Thus, for each ligand docked into each CAII structure, 100 IFP strings were generated. These strings were then compared to the CAII-rIFP by using the Tanimoto similarity index, Tc-IFP, defined as:

$$\mathrm{Tc}-\mathrm{IFP}=\frac{\left|A\cap B\right|}{\left|A\cup B\right|}$$

where A ∩ B is the number of switched-on bits common to the fingerprint strings A and B and A∪B is the sum of them. Tc-IFP calculated between the CAII-fingerprints of a pose and CAII-rIFP was used as a scoring function for evaluating the quality of the pose. Among the resulting 100 docking poses generated for each ligand, the one possessing the highest Tc-IFP was considered as the best-ranked pose and its RMSD with respect to the experimental ligand binding disposition was used for the statistical analysis.

### 3.6. Clustering of the CAII Inhibitors

The ligand–protein IFP string previously generated for each ligand-CAII X-ray complex (see above) was compared to all other IFP strings by measuring the Tc-IFP. On this basis a 127 × 127 matrix was generated reporting the different Tc-IFP values. The 127 co-crystal structures were then clustered based on the obtained Tc-IFP values so that all the ligands with similar ligand–protein IFPs were grouped together. For doing this calculation an in-house program already applied for ligand pose clustering in our previous studies [15,28] was used. The clustering algorithm employed by this program was the complete-linkage method, which is an agglomerative type of hierarchical clustering. This method starts considering each element in a cluster of its own. The clusters are then sequentially combined into larger ones, until all elements are in the same cluster. At each step, the two clusters separated by the shortest distance are combined. We selected a Tc-IFP clustering threshold of 0.40; therefore, the so obtained clusters contained the group of ligands which have a similarity score higher than 0.40 with respect to all other ligands belonging to the same cluster.

## 4. Conclusions

Carbonic anhydrase II is considered as an extremely important target for the development of diuretics, anti-osteoporosis, anti-glaucoma, and anti-tumor drugs. Therefore, the development of novel CAII inhibitors is a hot topic in medicinal chemistry. Despite the undeniable usefulness of ligand-protein docking in structure-based drug design of enzyme inhibitors, classic docking procedures often fail in predicting reliable ligand binding modes within a given receptor and novel procedures are needed to allow higher accuracy in the identification of the most energetically favored disposition of a ligand into its target protein [16]. Based on this consideration, we first performed cross-docking studies to thoroughly analyze the reliability of Autodock4 software in predicting the binding modes of potent CAII inhibitors within the catalytic site of the enzyme. Then, based on the main ligand–protein interactions detected in the CAII-inhibitor co-crystal structures of the most active CAII ligands, we generated ligand–protein interaction fingerprints (IFPs) that were used to develop an efficient and reliable post-docking procedure, which was able to identify native-like binding poses of CAII inhibitors better than Autodock4 scoring function. Precisely, the use of our IFP-based scoring function allowed the prediction of docking solutions with an aRMSD about 30% lower than that obtained by using Autodock4 scoring function and increased about 13% the number of reliable docking solutions. Overall, our analysis highlighted some interesting points: a) Autodock4 algorithm would have the potential of predicting highly reliable binding modes for CAII ligands; b) the scoring function implemented in Autodock4 limits the qualitative performance of the software with respect to the ideal performance allowed by an optimal scoring function able to always rank the most reliable docking solution as the top-scored pose; c) our scoring function based on the comparison of IFPs is able to better exploit the potential of Autodock4 search engine and to calibrate it for the prediction of CAII inhibitors’ binding modes; and d) our IFP-based scoring function showed to be a useful tool for the binding mode analysis of CAII inhibitors and for identifying key interactions shared by structurally dissimilar ligands. Based on these considerations, we envision that the application of our IFP-based scoring function would be similarly effective in improving the reliability of other docking software in the pose prediction of CAII ligands’ binding dispositions. Moreover, its application in structure-based virtual screening studies would be beneficial for the identification of novel CAII inhibitors, due to the possibility of ranking compounds in a knowledge-based way (i.e., prioritizing those showing the same key interactions with CAII observed for the reference inhibitors), but still allowing a considerable structural diversity among the prioritized hits. For instance, the application of our approach for docking and post-processing a large database of compounds into CAII binding site would not only improve the reliability of the binding poses predicted for the compounds but it would also provide a qualitative tool to select the most promising ligands. In fact, the docked molecules could be filtered based on their IFPs selecting only those showing a high Tc-IFP score, which will thus present in their predicted binding mode a high number of ligand–protein interactions shared by the most potent CAII inhibitors. Our approach could be then combined with other structure-based methods like molecular dynamics, which could be used to evaluate the stability of such ligand–protein interactions [29,30]. Despite the IFP-based approach showing good results in the reliability analysis performed with the external data sets of CAII ligands, it is worth considering that our method will inevitably suffer from the limitations typical of all knowledge-based approaches. Precisely, a molecule able to bind CAII, thanks to a pattern of ligand–protein interactions considerably different from those formed by the 127 inhibitors used for the training set, will show a low Tc-IFP score, since the reference CAII fingerprint (CAII-rIFP) does not take into account such interactions. However, since the 44 residues whose interactions are considered by the CAII-rIFP cover almost the whole ligand accessible surface of the enzyme in the proximity of its catalytic zinc ion (see Figure S4 in the Supporting Information), we are quite confident that this eventuality is not very likely to happen, with the only exception of allosteric ligands. Another typical limitation can be encountered in the application of our approach for a different protein target. In fact, only if a considerable number of ligand–protein X-ray structures are available for that protein and can thus be used as training set, a reliable reference IFP can be developed. However, in case only few X-ray co-crystal structures of the desired target are available, the IFP approach could still be used to identify potential ligands able to form the same interactions observed for the few co-crystallized ligands, as a sort of pharmacophore-guided docking approach.

## Figures and Tables

**Table 1:** Schematic representation of the fingerprint table for the interaction of one ligand with one single residue.

Anum	Name and Number of the Residue (for Example R92)
0 or 1	H-bonds (acceptor)
0 or 1	H-bonds (donor)
0 or 1	Hydrophobic contacts
0 or 1	π—π stacking interaction
0 or 1	T-stacking interaction
0 or 1	Cation-π interaction
0 or 1	Salt bridge

## Supplementary Materials

Supplementary materials can be found at http://www.mdpi.com/1422-0067/19/7/1851/s1.
