# Supplementary material for: Development of a Fingerprint-Based Scoring Function for the Prediction of the Binding Mode of Carbonic Anhydrase II Inhibitors
Source: Int J Mol Sci. 2018 Jun 23;19(7):1851. doi: 10.3390/ijms19071851 (PMC6073570; doi:10.3390/ijms19071851)
Supplement: Supplementary file 1 [file ijms-19-01851-s001.pdf]

Supplementary: Development of a Fingerprint-Based Scoring Function for the Prediction of the Binding Mode of Carbonic Anhydrase II Inhibitors

Giulio Poli [1], Vibhu Jha [1], Adriano Martinelli [1], Claudiu T. Supuran [2], Tiziano Tuccinardi [1,*]

[1] Department of Pharmacy, University of Pisa, 56126 Pisa, Italy; giulio.poli@unipi.it (G.P.); vibhujha16@gmail.com (V.J.); adriano.martinelli@unipi.it (A.M.)
[2] NEUROFARBA Department, Sezione di Scienze Farmaceutiche e Nutraceutiche, Università degli Studi di Firenze, Sesto Fiorentino, 50019 Florence, Italy; claudiu.supuran@unifi.it
* Correspondence: tiziano.tuccinardi@unipi.it; Tel: +39-0502219595

Table of Contents

Figure S1. Fingerprint analysis for 3DBU Page 2
Figure S2. Tc-IFP similarity analysis of the deposited ligand-CAII X-ray complexes Page 3
Figure S3. Superimposition of the 127 CAII X-ray structures Page 4
Figure S4. Protein binding site mapped by the CAII-rIFP Page 5
Table S1. Structure and interactions of the 127 ligands co-crystallized with CAII Page 6
Table S2. Structures of the 70 ligands used as a first external test set Page 17
Table S3. Structures of the 15 ligands used as additional second external test set Page 21

**W005**	0	0	0	0	0	0	0
**Y007**	0	0	0	0	0	0	0
**F020**	0	0	0	0	0	0	0
**R 058**	0	0	0	0	0	0	0
**L060**	0	0	0	0	0	0	0
**N062**	0	0	0	0	0	0	0
**H064**	0	0	0	0	0	0	0
**A065**	0	0	0	0	0	0	0
**F066**	0	0	0	0	0	0	0
**Q067**	0	0	0	0	0	0	0
**E069**	0	0	0	0	0	0	0
**D072**	0	0	0	0	0	0	0
**I091**	0	0	0	0	0	0	0
**Q092**	0	1	0	0	0	0	0
**F093**	0	0	0	0	0	0	0
**H094**	0	0	0	0	1	1	0
**F095**	0	0	0	0	0	0	0
**H096**	0	0	0	0	0	0	0
**H119**	0	0	0	0	0	0	0
**V121**	0	0	0	0	0	0	0
**H122**	0	0	0	0	0	0	0
**W123**	0	0	0	0	0	0	0
**D129**	0	0	0	0	0	0	0
**F130**	0	0	1	0	1	0	0
**G131**	0	0	0	0	0	0	0
**K132**	0	0	0	0	0	0	0
**A133**	0	0	0	0	0	0	0
**V134**	0	0	0	0	0	0	0
**Q135**	0	0	0	0	0	0	0
**L140**	0	0	0	0	0	0	0
**V143**	0	0	0	0	0	0	0
**S196**	0	0	0	0	0	0	0
**L197**	0	0	1	0	0	0	0
**T198**	0	1	0	0	0	0	0
**T199**	0	1	0	0	0	0	0
**P200**	0	0	0	0	0	0	0
**P201**	0	0	0	0	0	0	0
**L202**	0	0	0	0	0	0	0
**L203**	0	0	0	0	0	0	0
**C205**	0	0	0	0	0	0	0
**V206**	0	0	0	0	0	0	0
**W208**	0	0	0	0	0	0	0
**N243**	0	0	0	0	0	0	0

Figure S1. Fingerprint analysis of the interaction between 5-[(phenylsulfonyl)amino]-1,3,4-thiadiazole-2-sulfonamide and CAII (PDB code 3DBU).

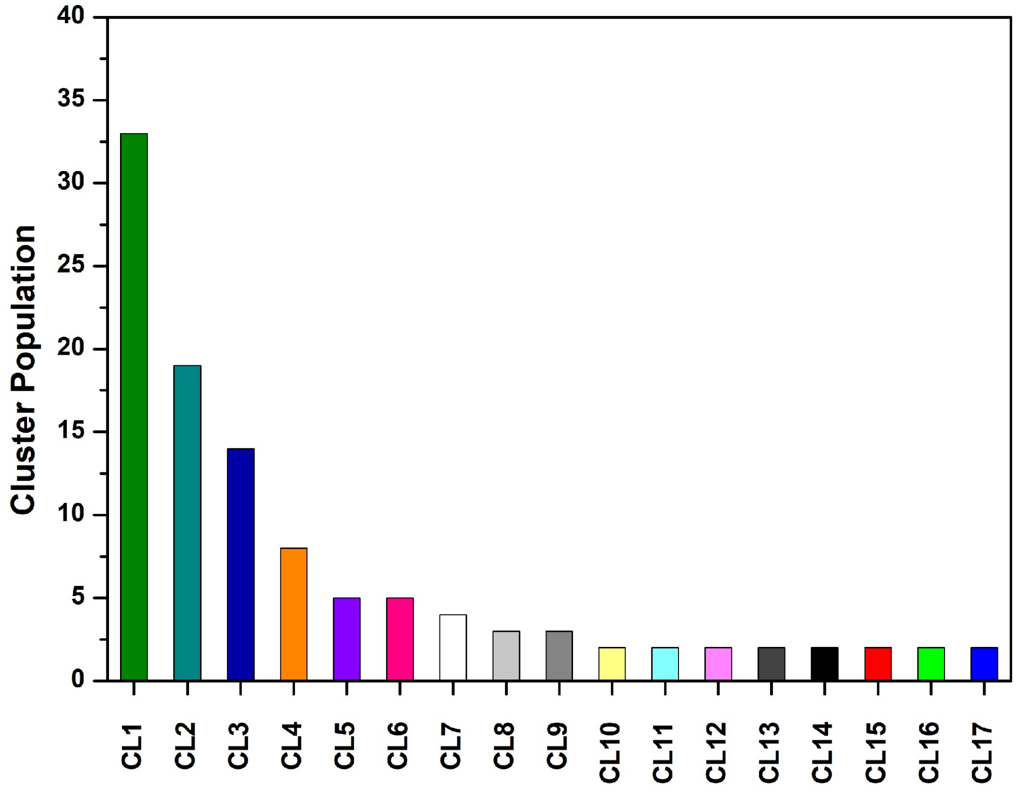

Figure S2. Similarity analysis of the deposited ligand-CAII X-ray complexes on the basis of the Tc-IFP score.

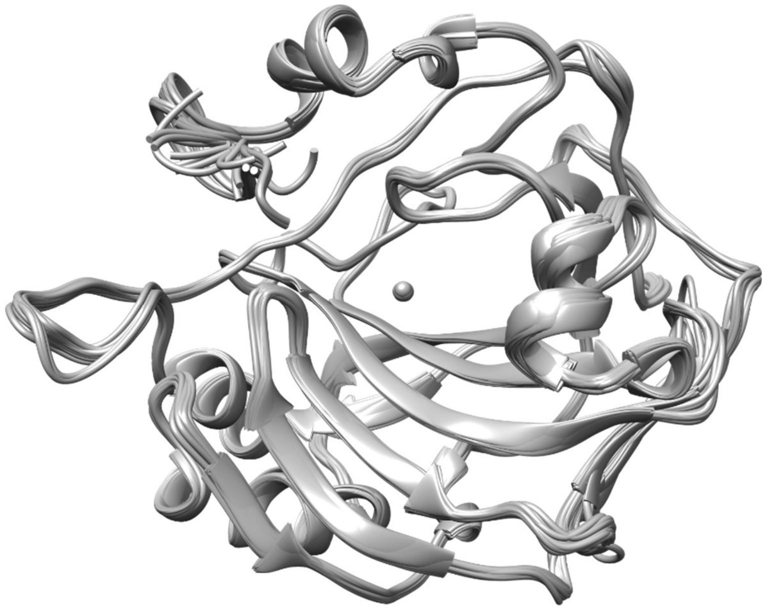

Figure S3. Superimposition of the 127 CAII X-ray structures.

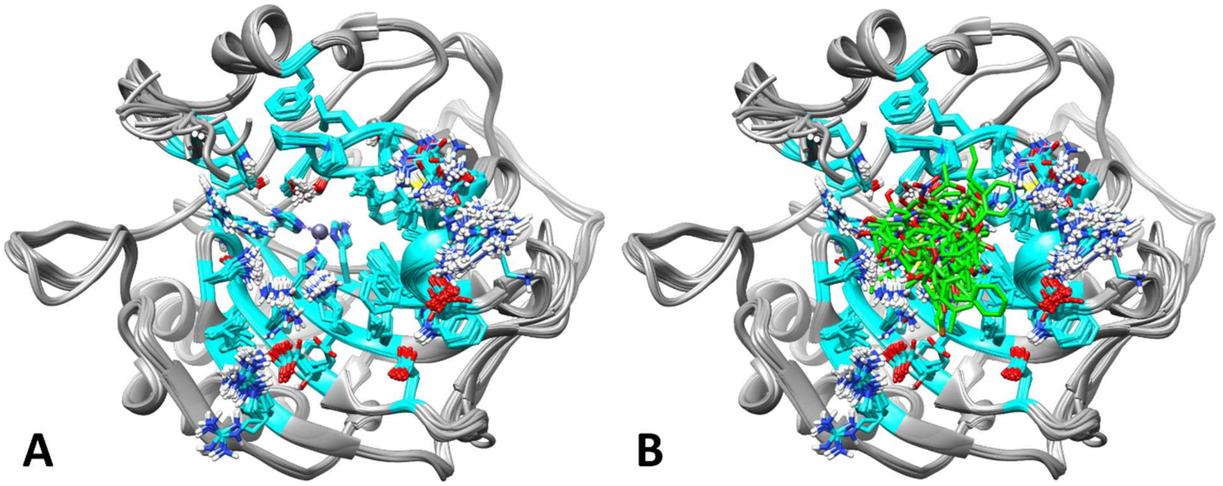

Figure S4. Protein binding site mapped by the CAII-rIFP. (A) The 44 residues of the 127 CAII X-ray structures whose interactions are mapped by the CAII-rIFP are showed in cyan sticks, while the co-crystallized ligands are hidden. (B) The 127 co-crystallized ligands are also shown in green sticks.

Table S1. Structure and interactions of the 127 ligands co-crystallized with CAII.

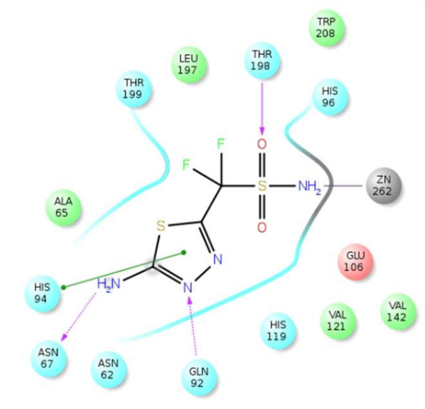	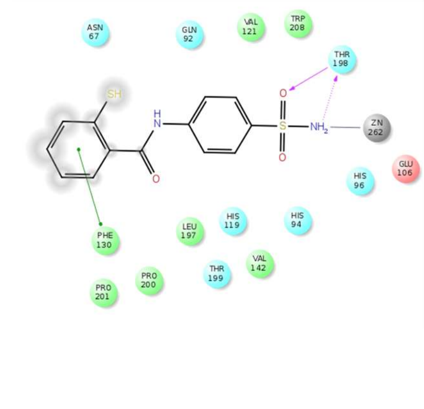	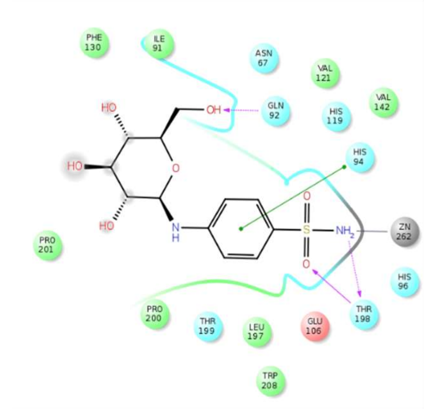
**2eu3**	**2hd6**	**2hl4**
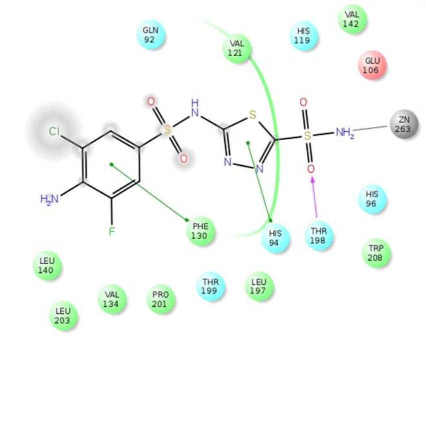	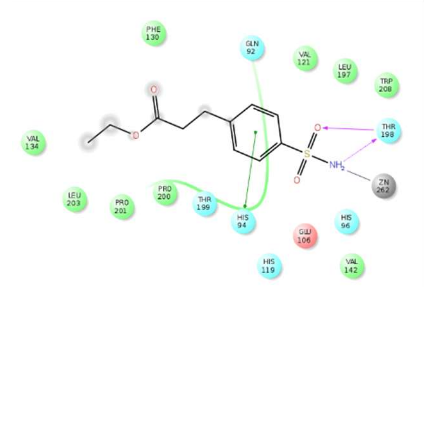	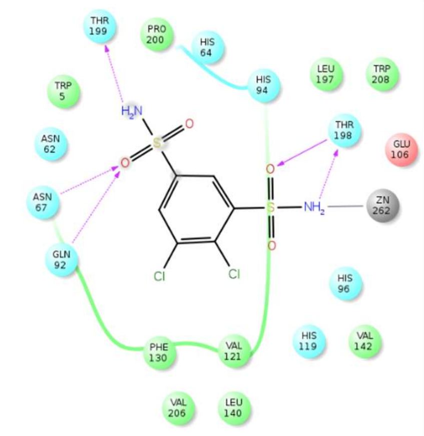
**2hoc**	**2nnv**	**2pou**
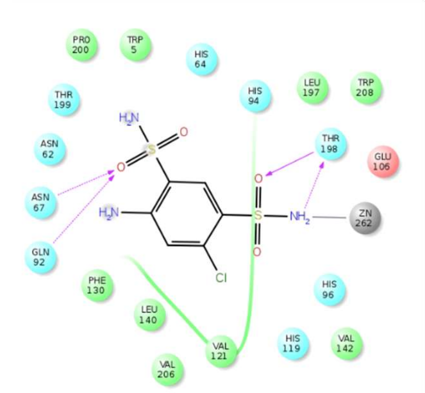	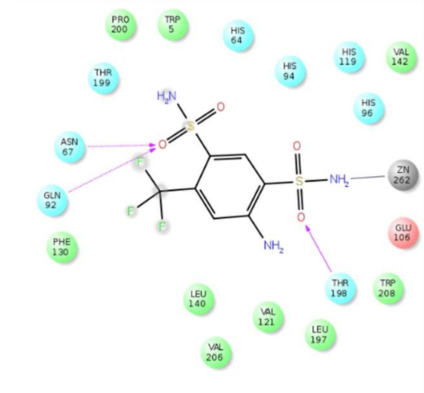	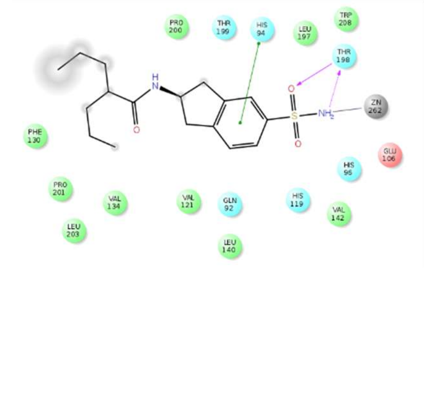
**2pov**	**2pow**	**2qo8**
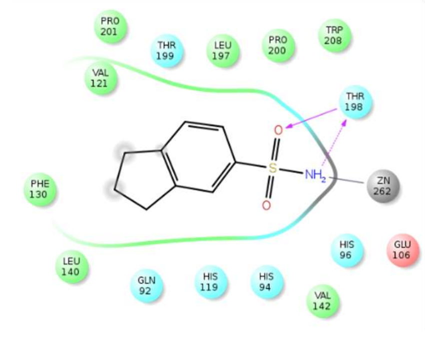	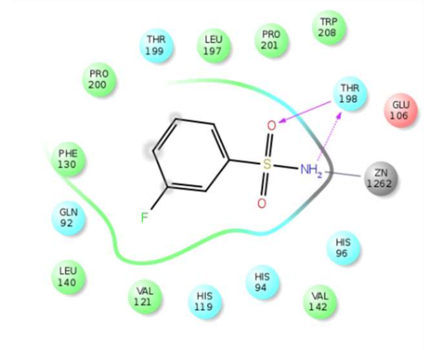	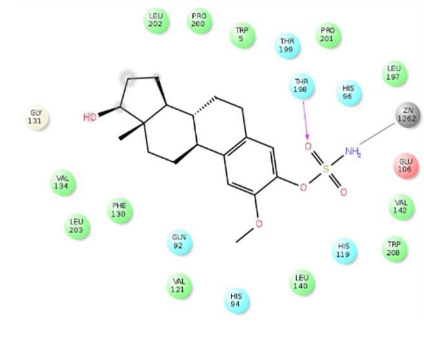
2qoa	2weo	2x7u

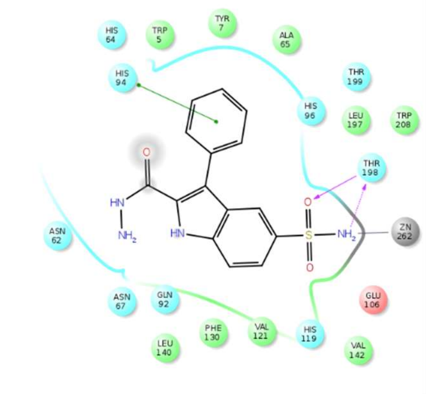	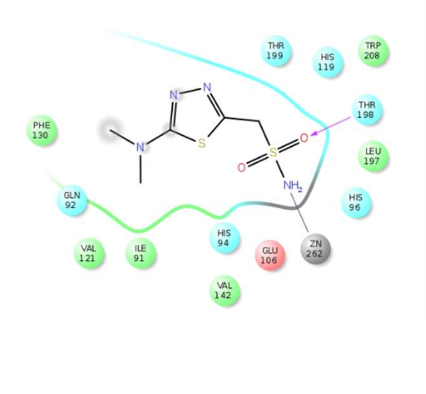	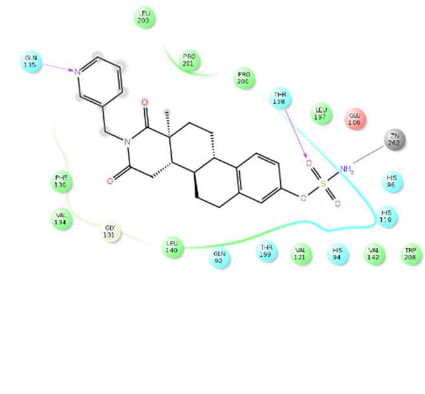
3b4f	3bl0	3c7p
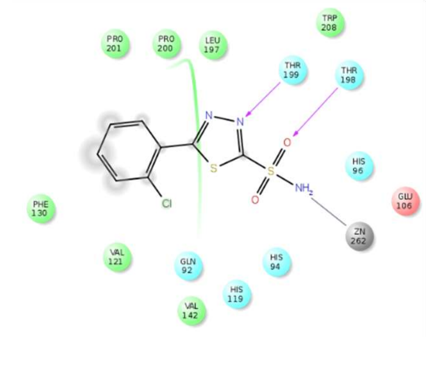	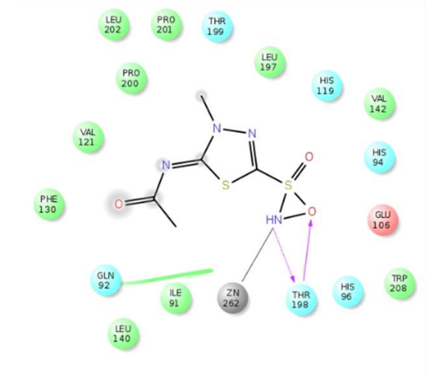	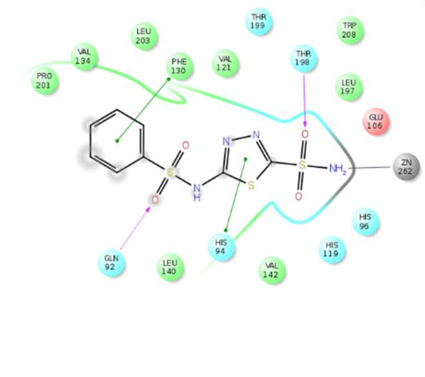
3d9z	3daz	3dbu
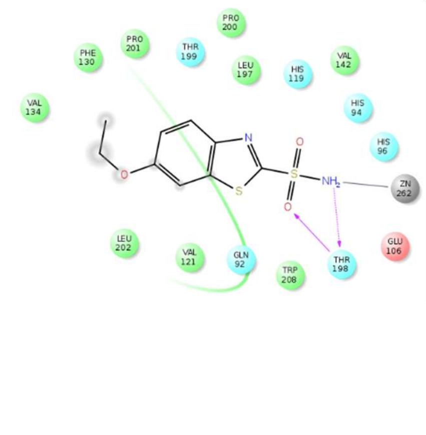	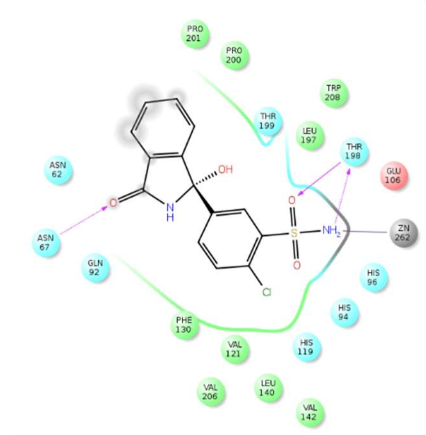	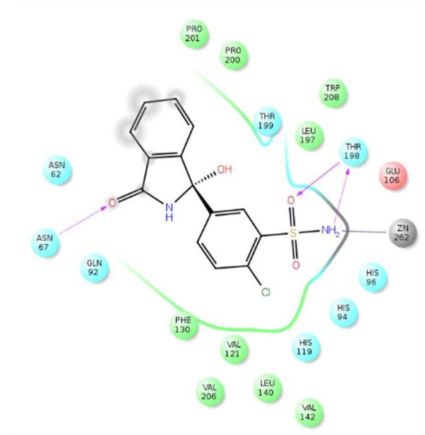
3dd0	3f4x	3f4x
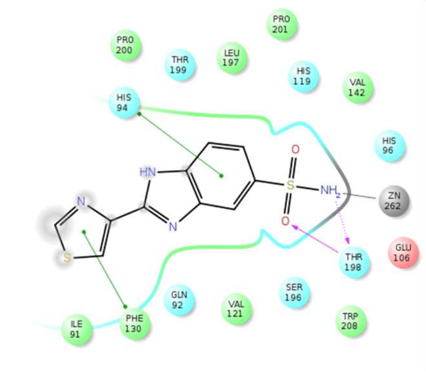	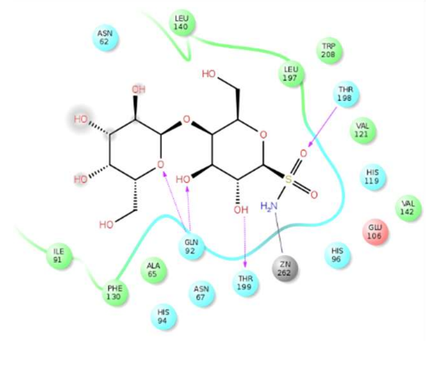	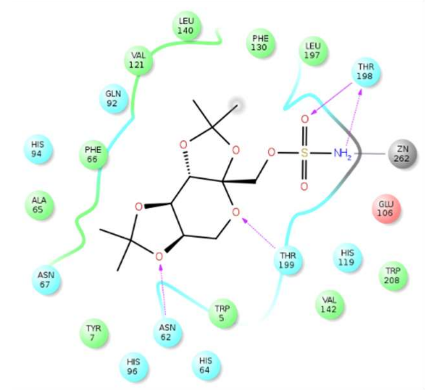
3ffp	3hkt	3hku

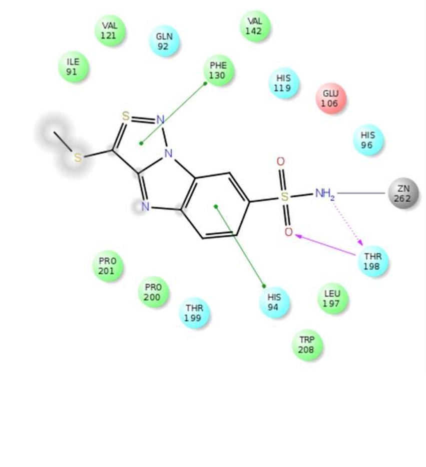

3hlj

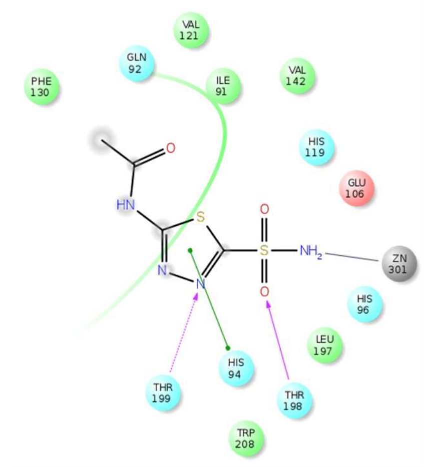

3hs4

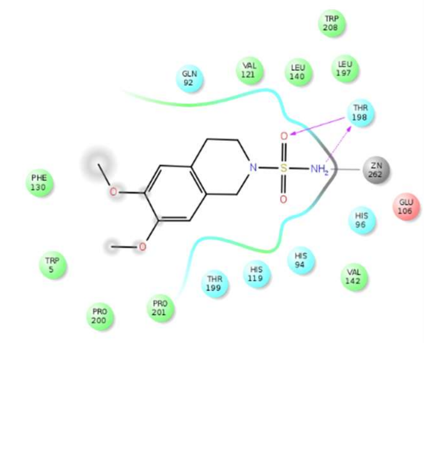

3igp

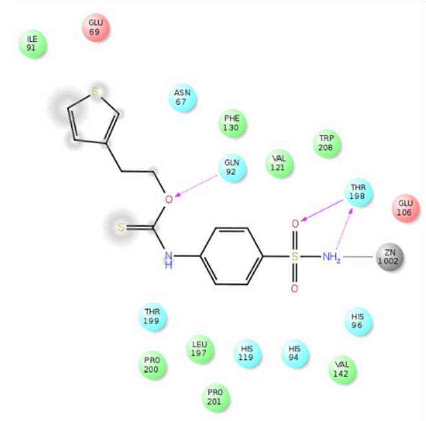

3k34

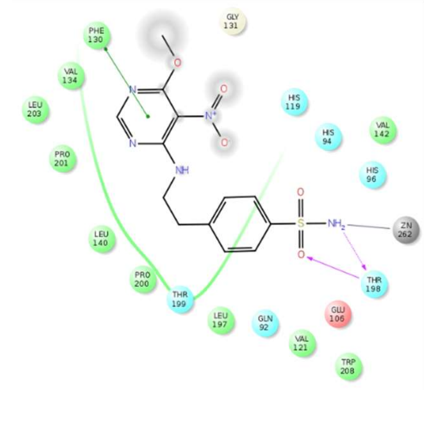

3m3x

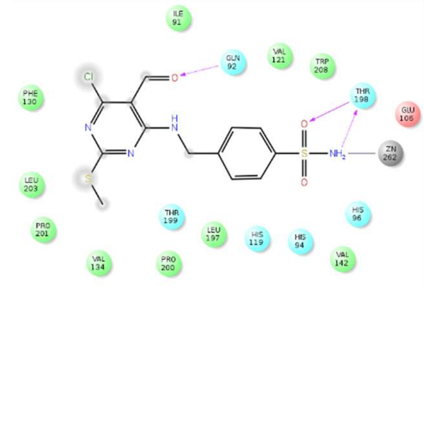

3m5e

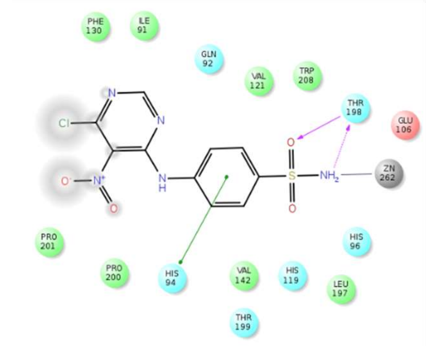

3m40

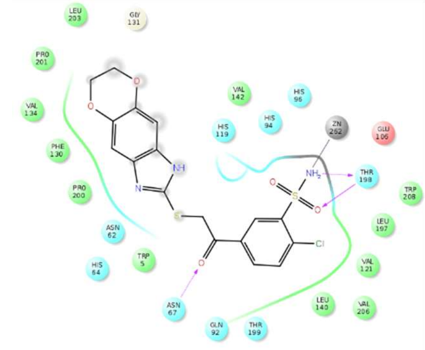

3m67

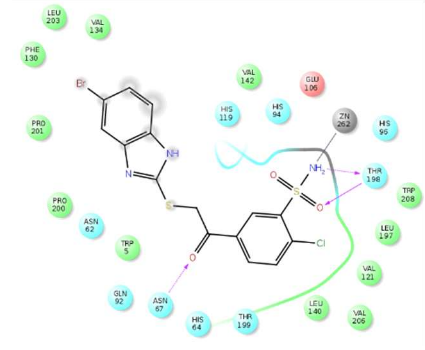

3m96

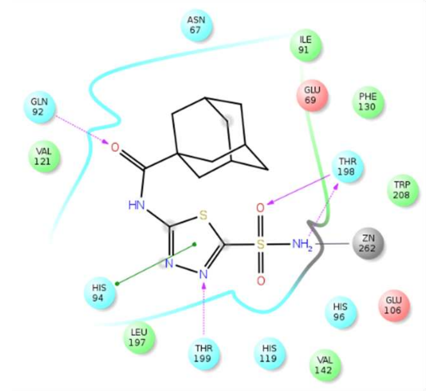

3mhc

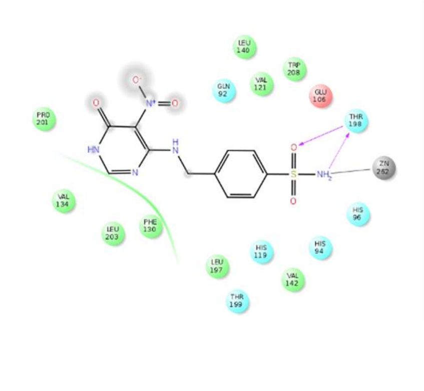

3mhi

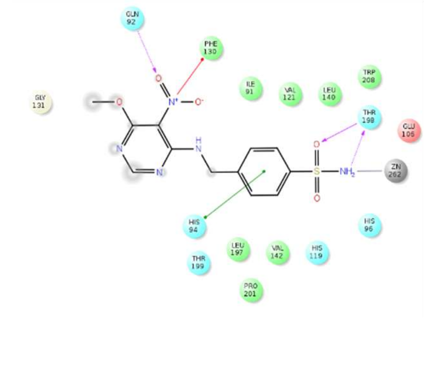

3mhl

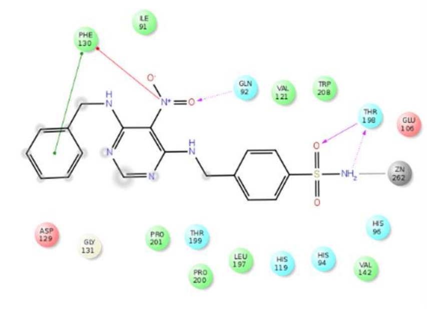	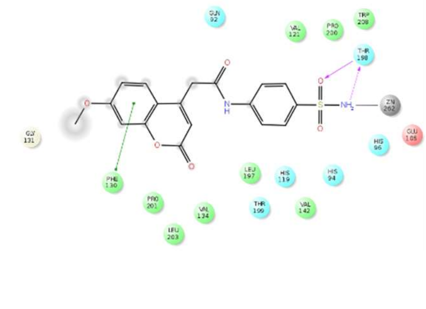	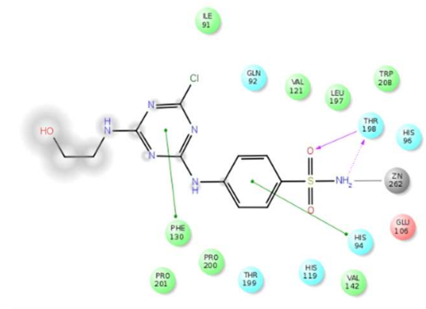
3mhm	3ml2	3mmf
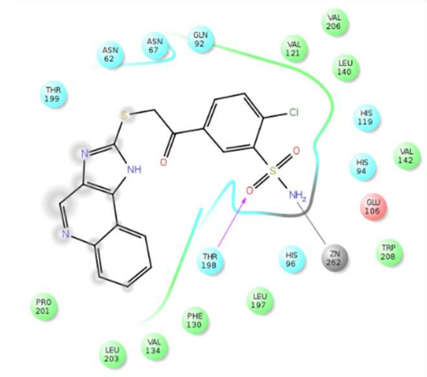	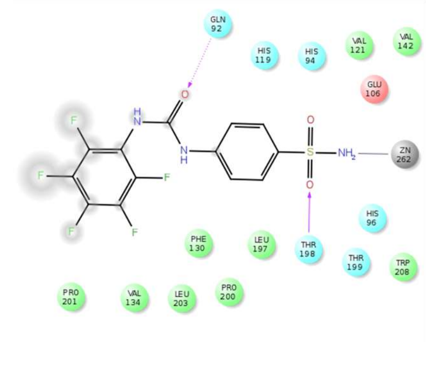	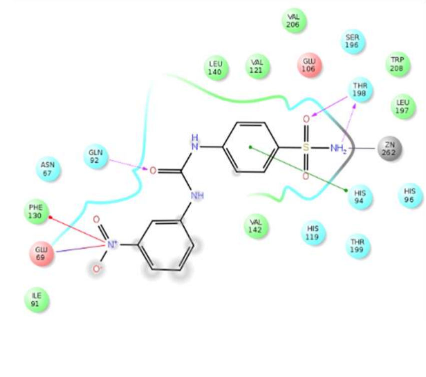
3myq	3n0n	3n2p
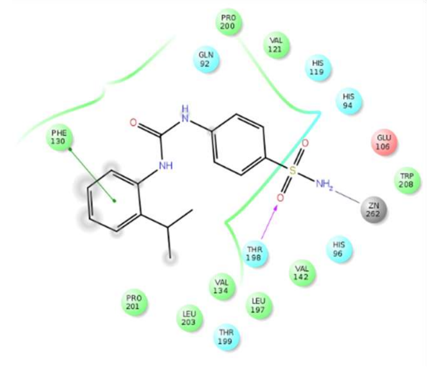	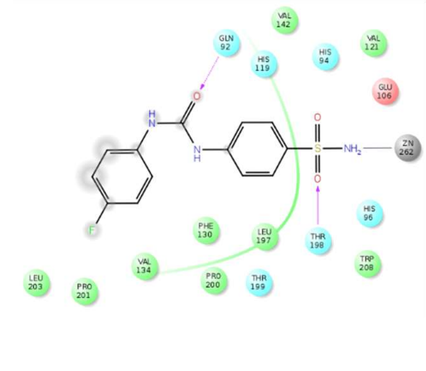	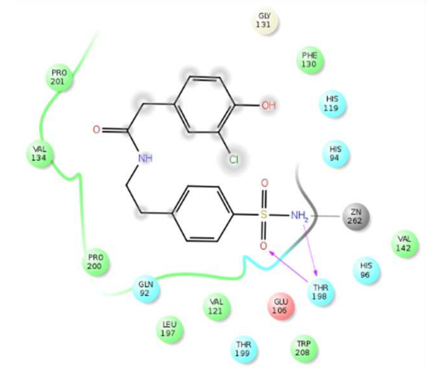
3n3j	3n4b	3nb5
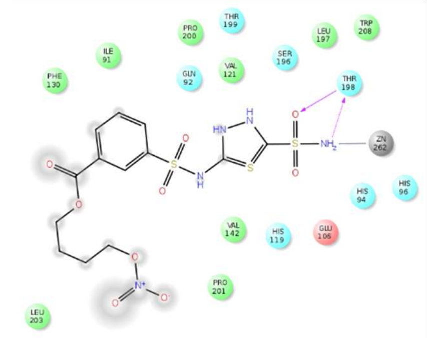	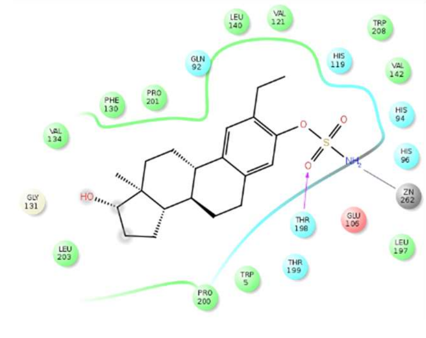	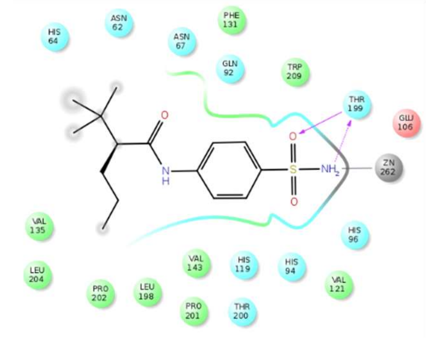
3ni5	3oim	3oy0

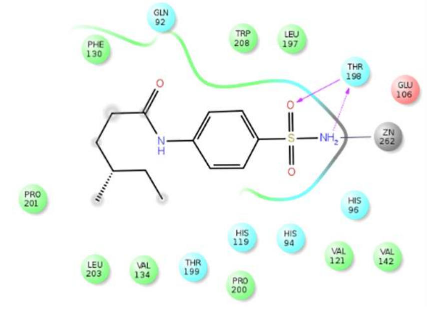	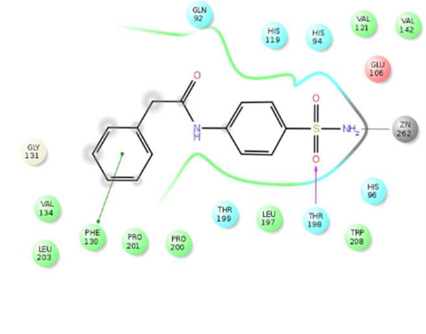	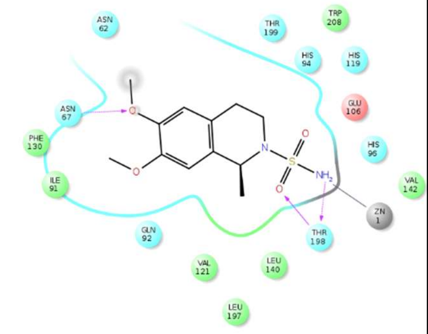
3oyq	3oys	3po6
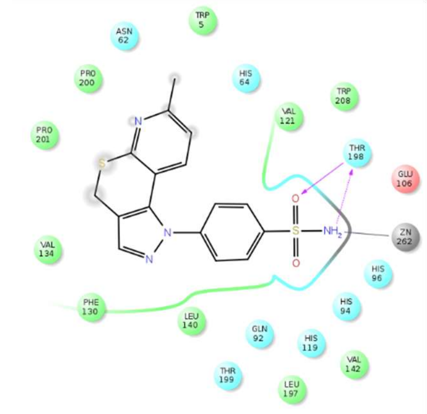	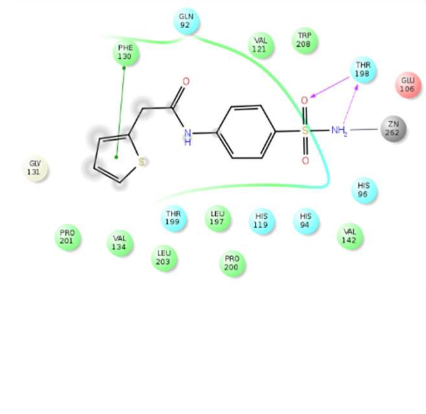	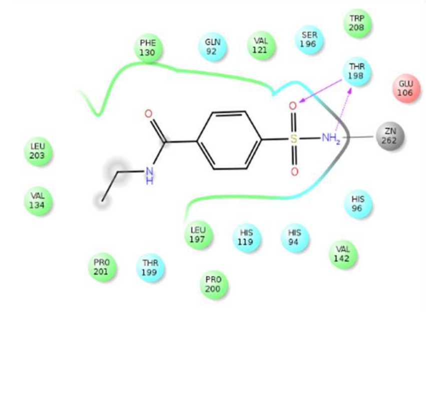
3qyk	3r16	3ryv
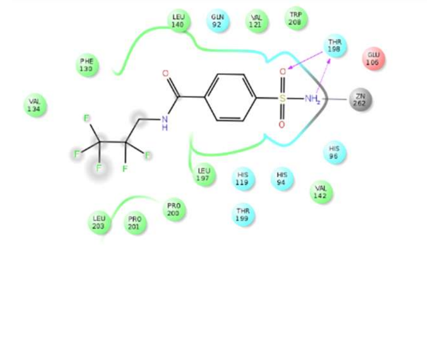	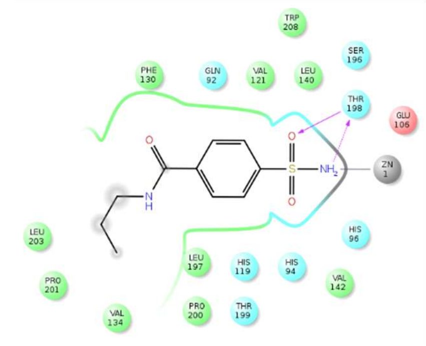	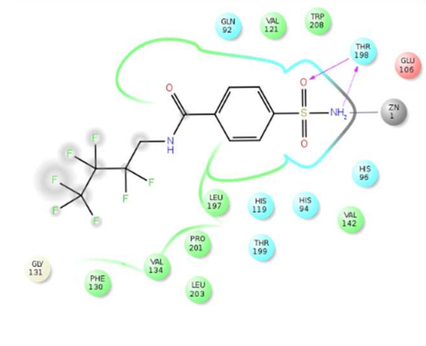
3ryx	3ryy	3ryz
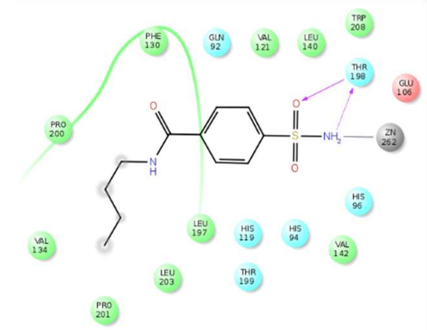	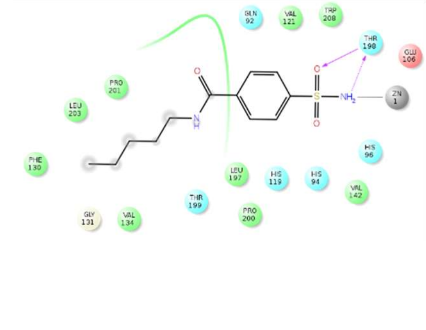	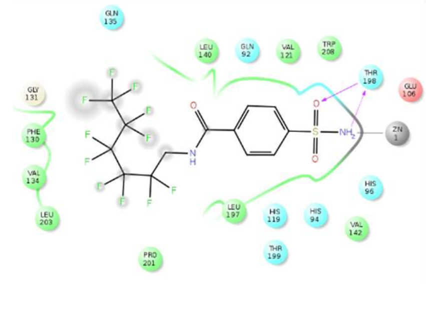
3rz0	3rz5	3rz7

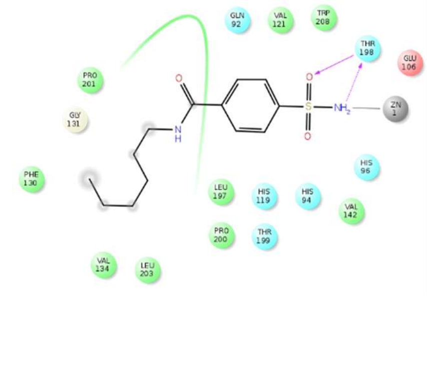

3rz8

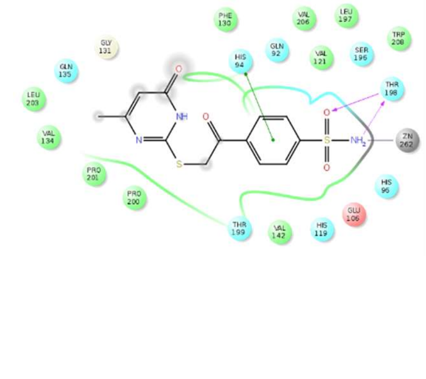

3s8x

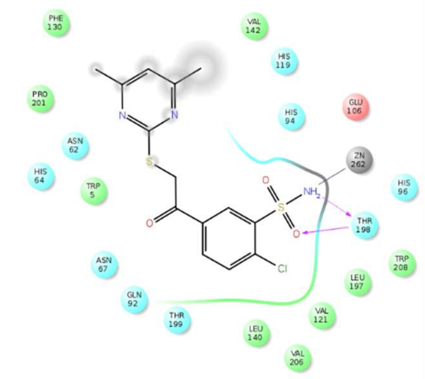

3s9t

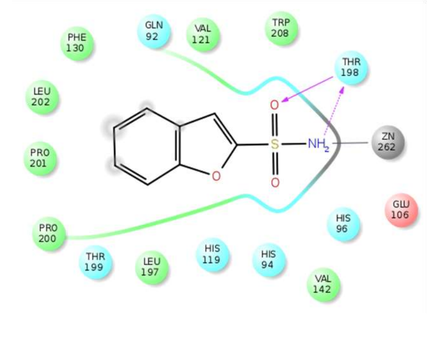

3s71

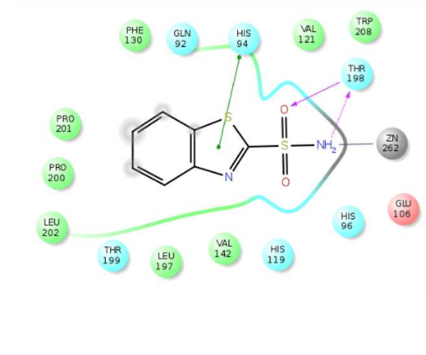

3s73

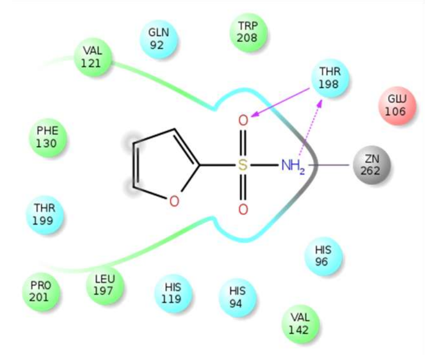

3s75

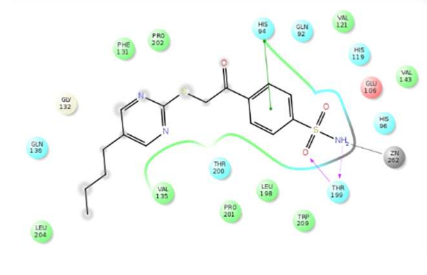

3sap

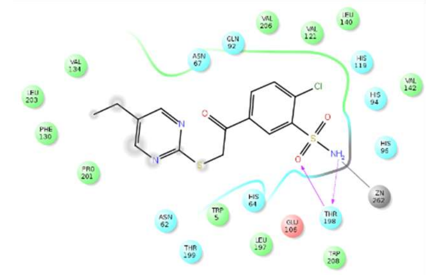

3sax

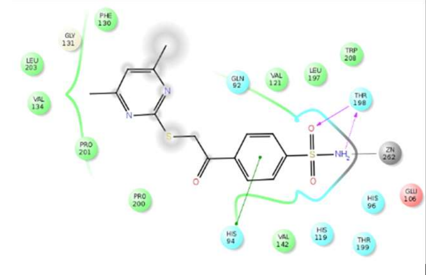

3sbh

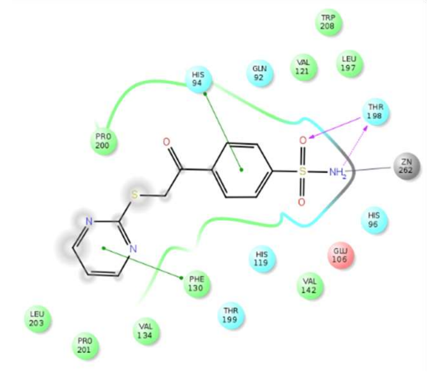

3sbi

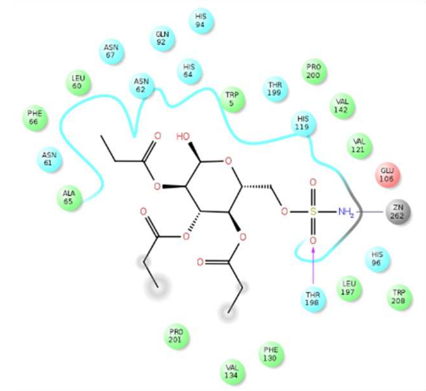

3t83

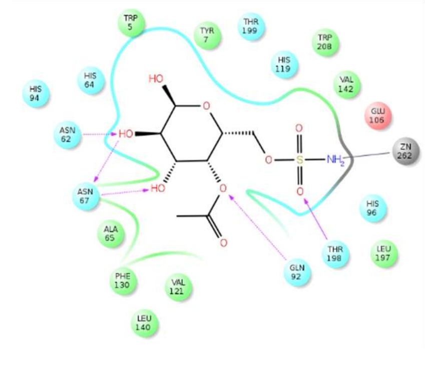

3t84

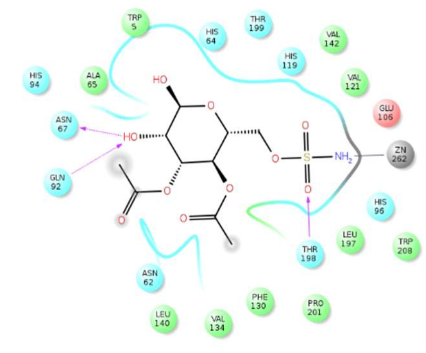	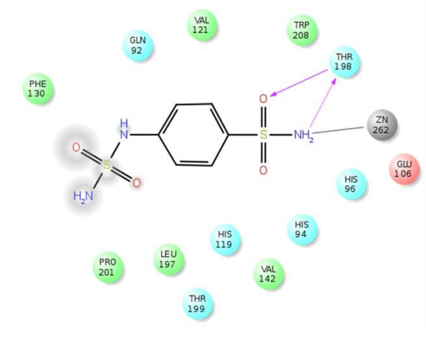	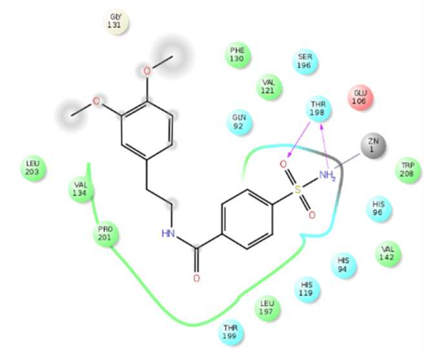
3t85	3v5g	3v7x
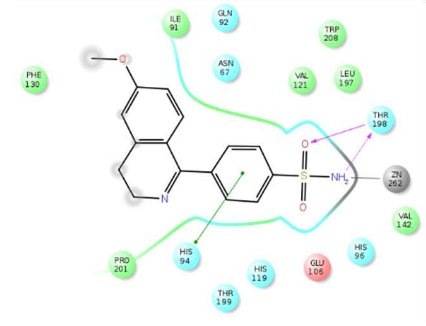	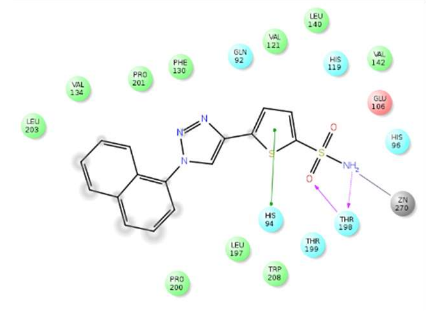	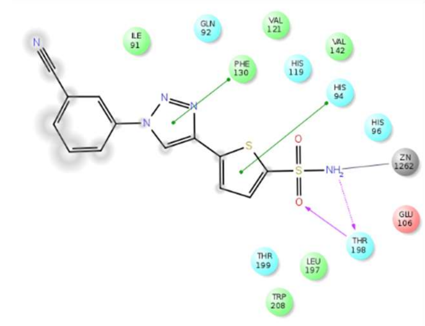
3vbd	4fb1	4bf6
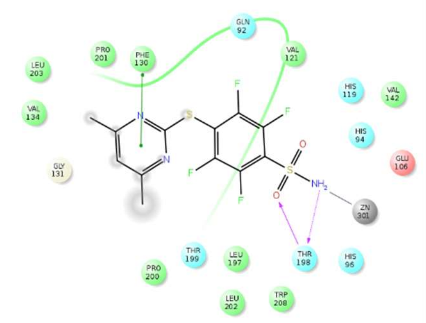	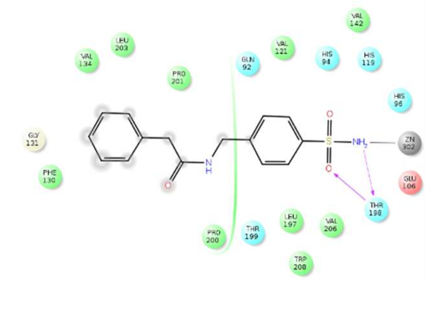	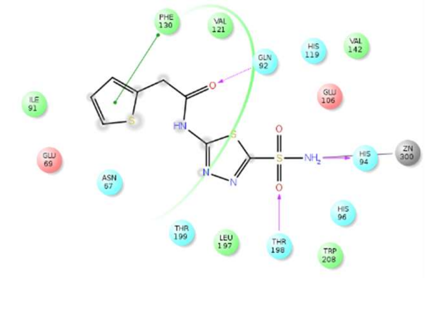
4ht0	4itp	4iwz
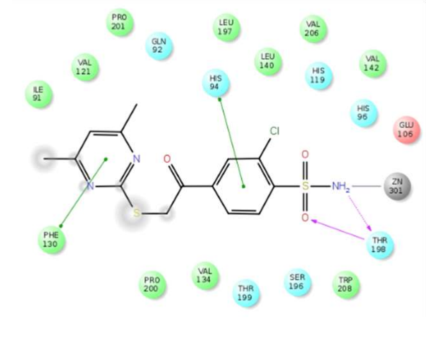	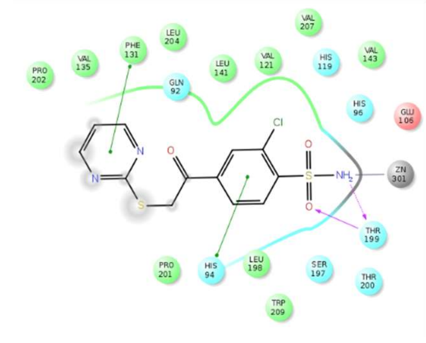	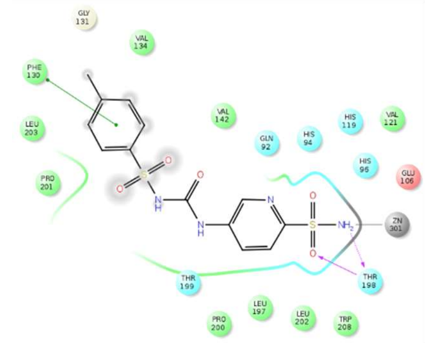
4kni	4knj	4kv0

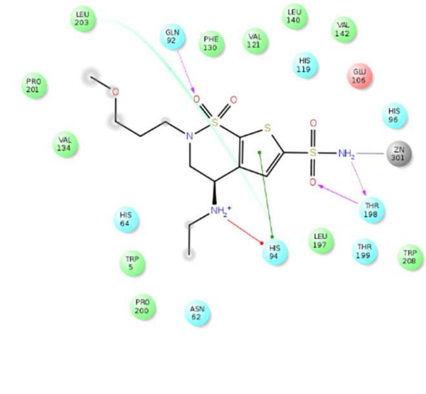	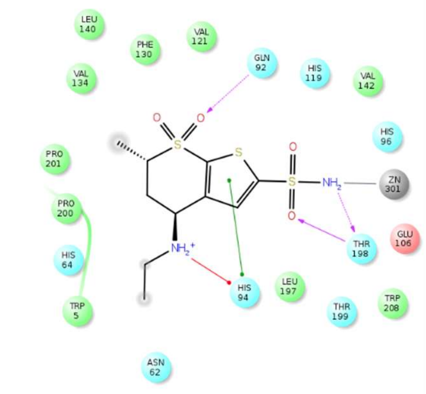	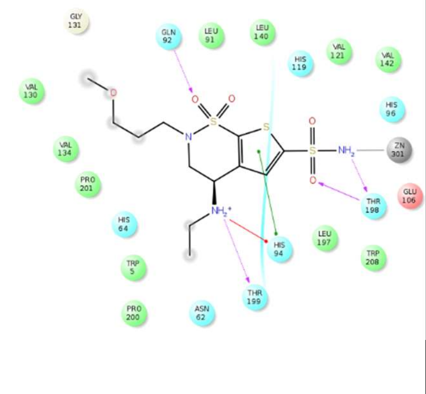
4m2r	4m2u	4m2v
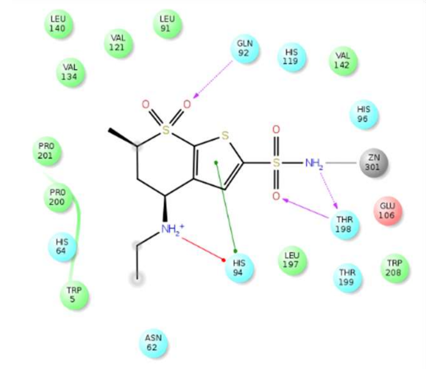	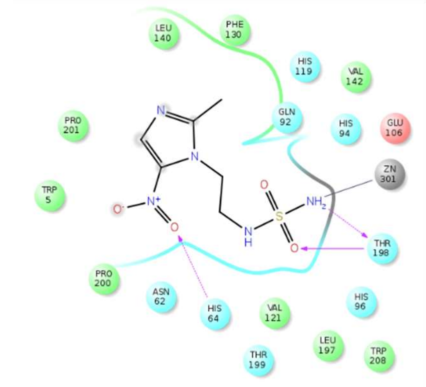	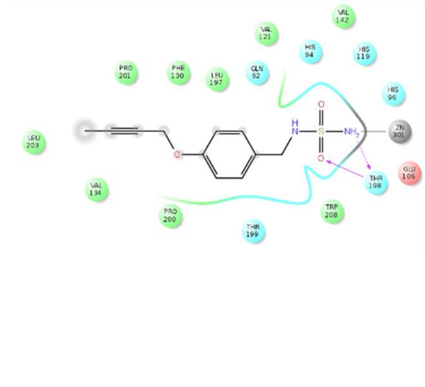
4m2w	4mo8	4pq7
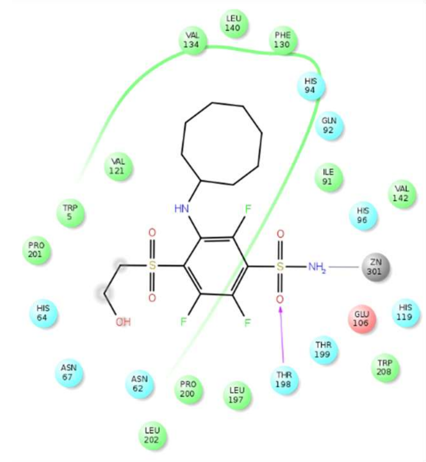	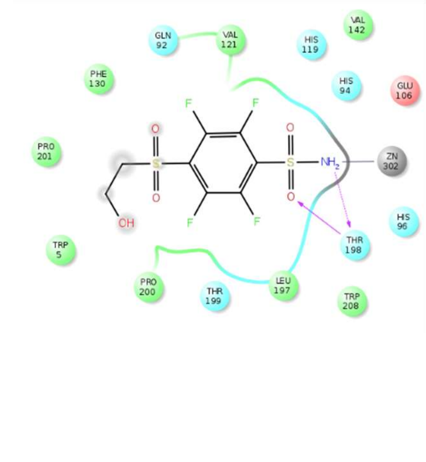	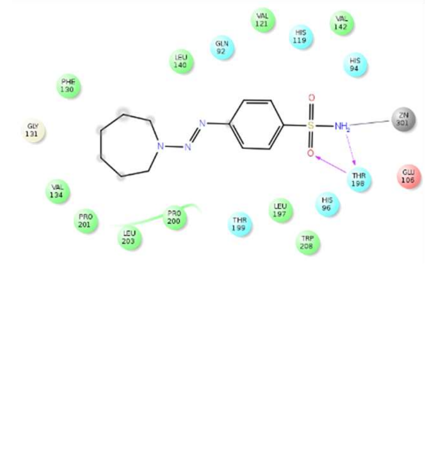
4pyy	4pzh	4q6d
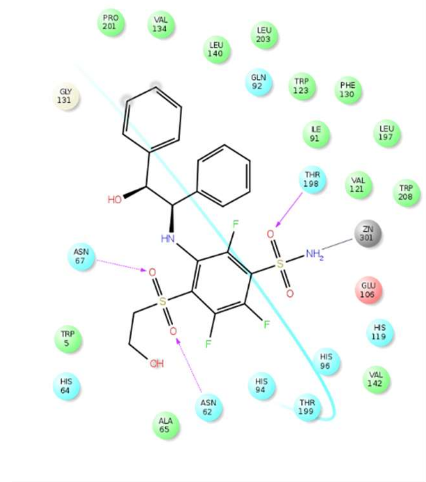	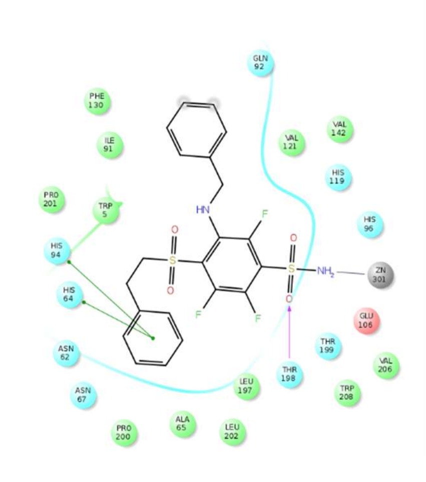	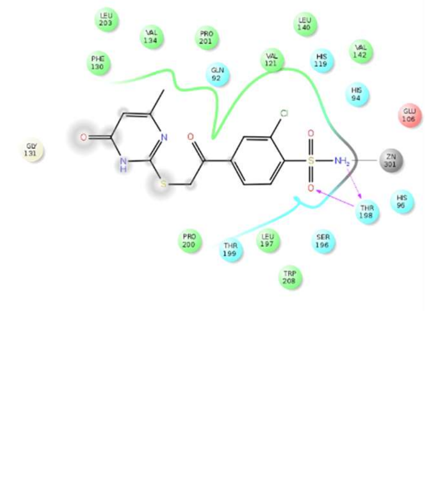
4qiy	4qjm	4qsa

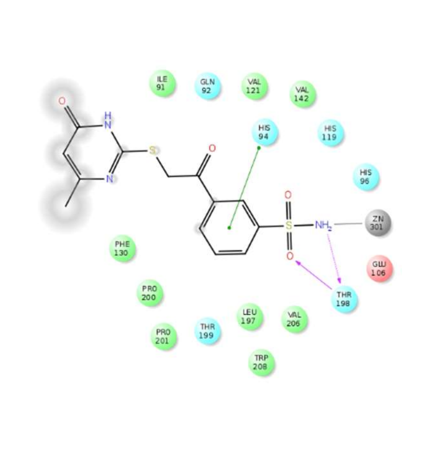	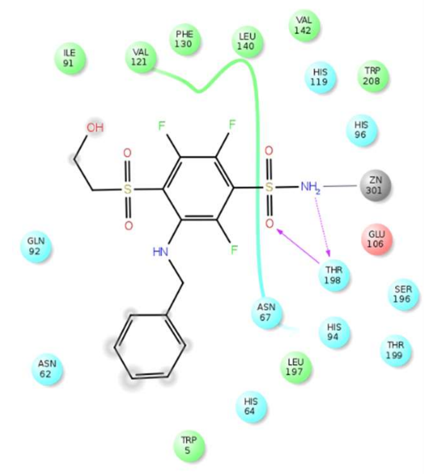	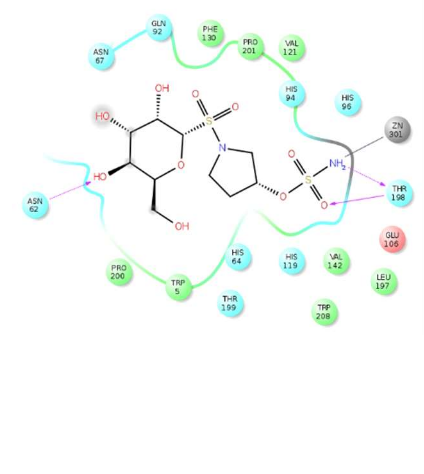
4qsb	4qtl	4r5b
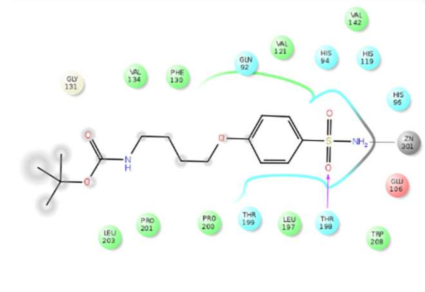	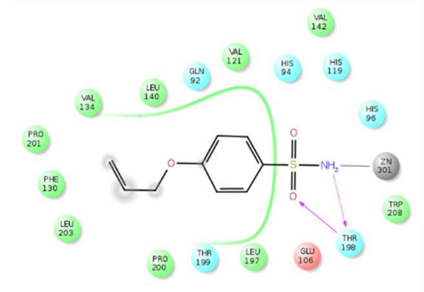	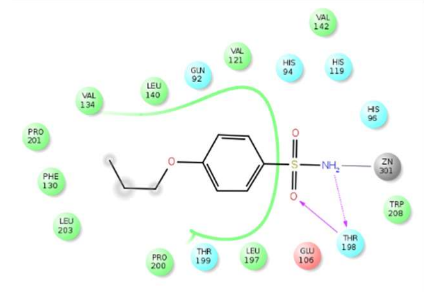
4rfc	4rux	4ruy
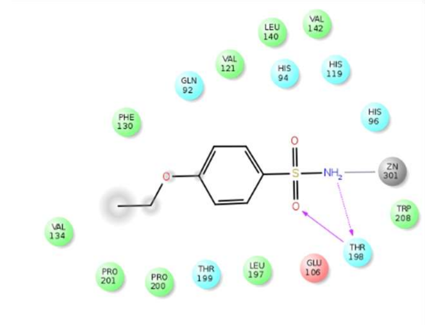	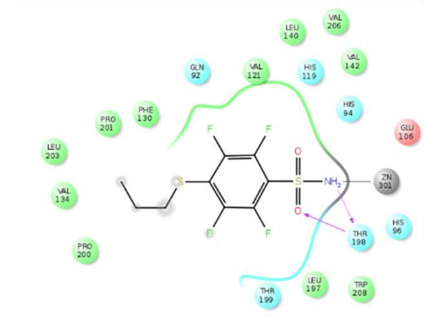	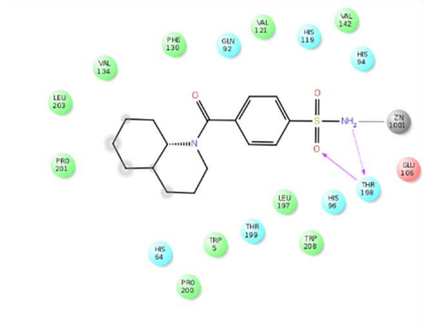
4ruz	4ww6	4z0q
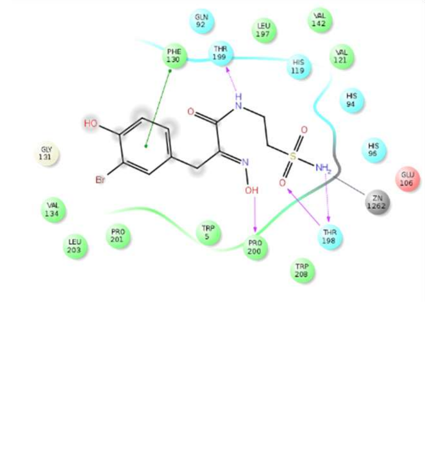	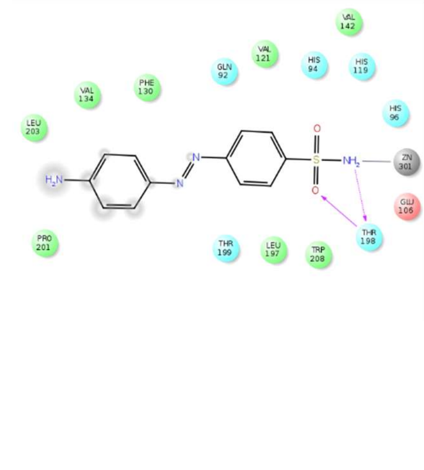	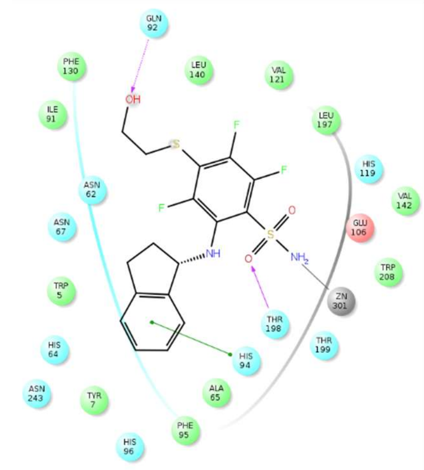
5a6h	5byi	5doh

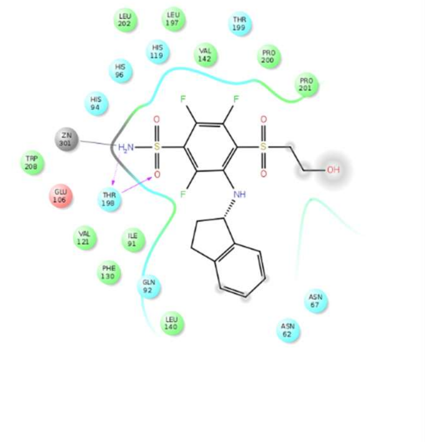	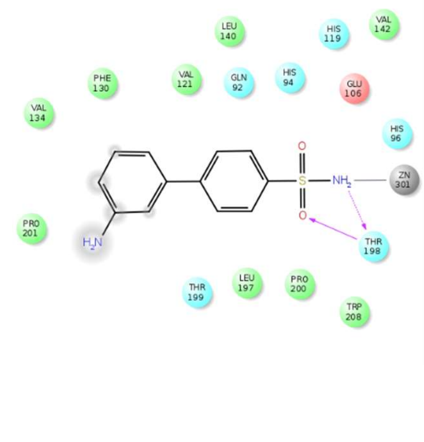	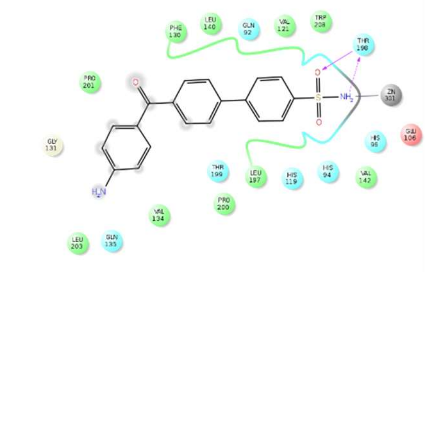
5drs	5e2k	5e2r
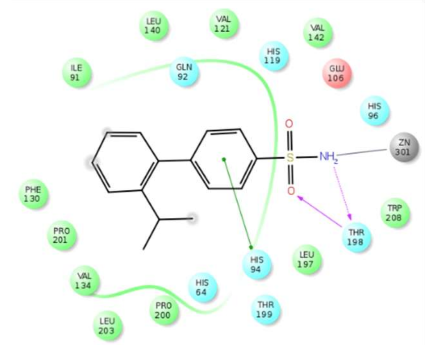	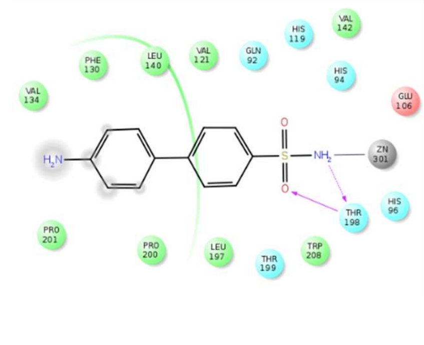	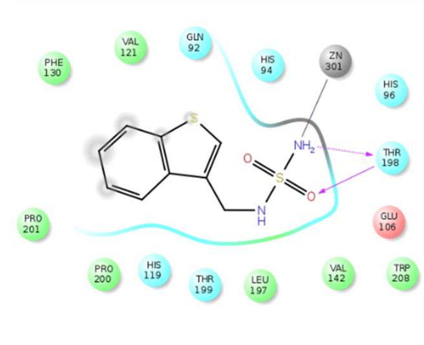
5e2s	5e28	5fdc
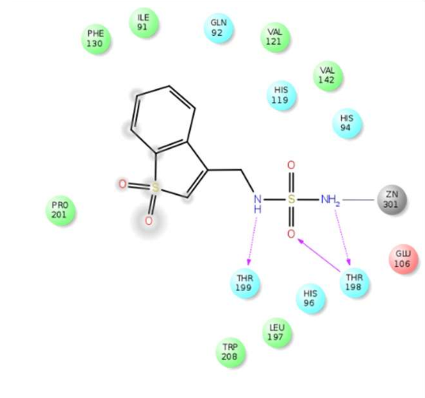	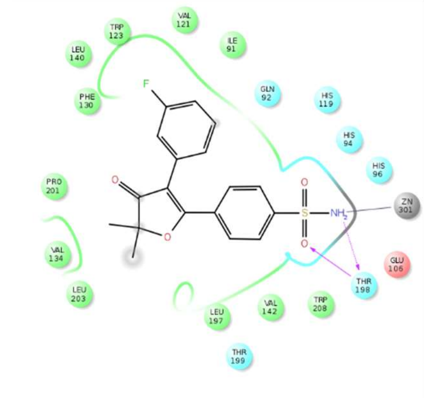	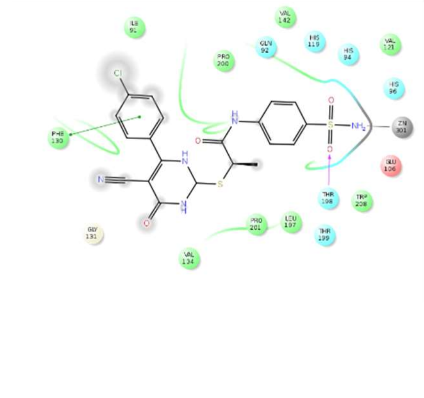
5fdi	5gmn	5j8z
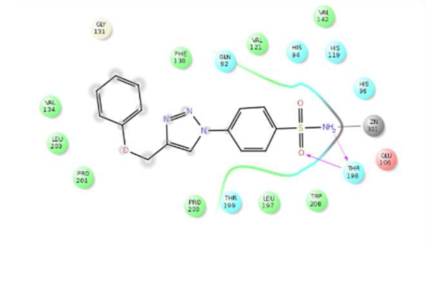	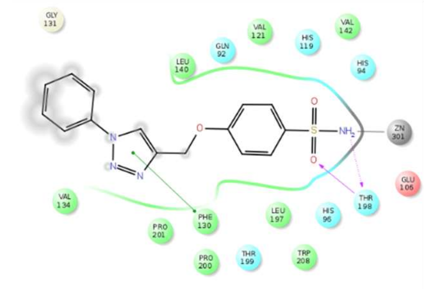	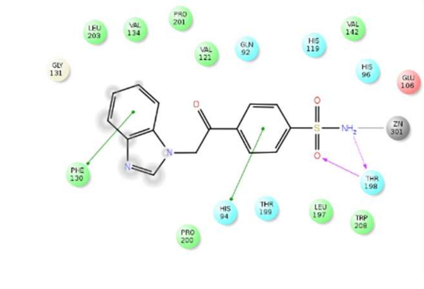
5jlq	5ljt	5ll4

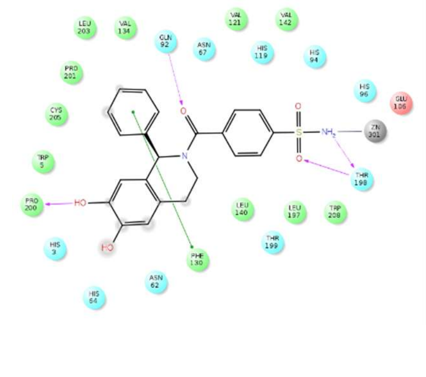	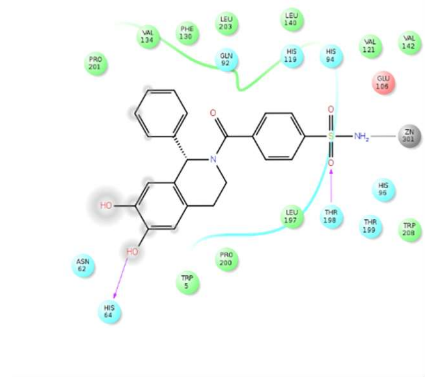	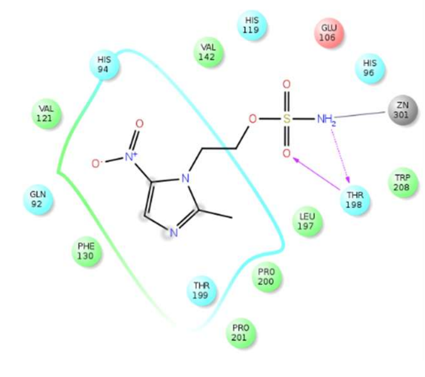
5n0d	5n0e	5o07
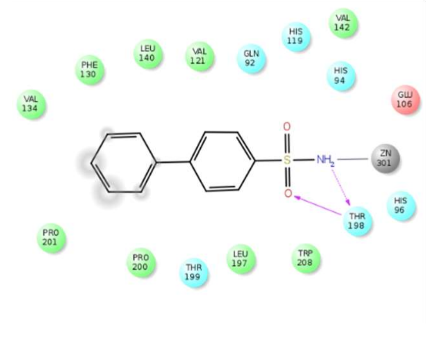	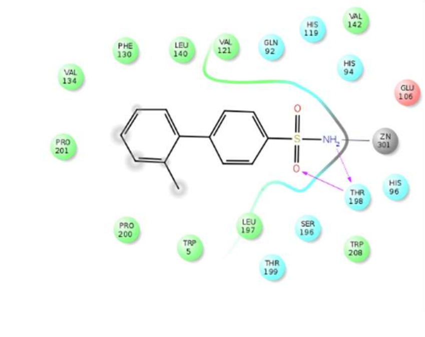	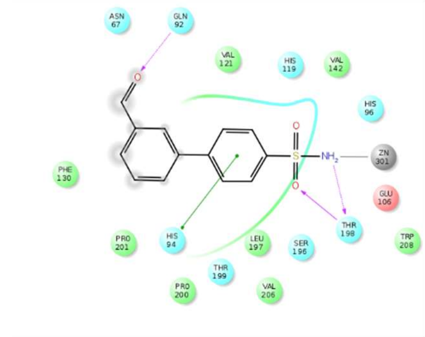
5sz0	5sz1	5sz2
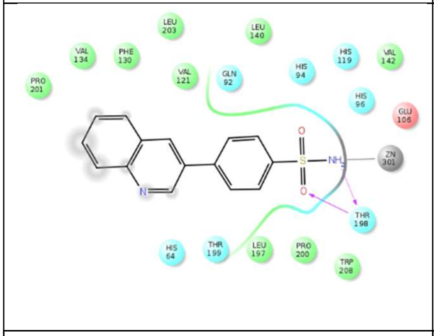	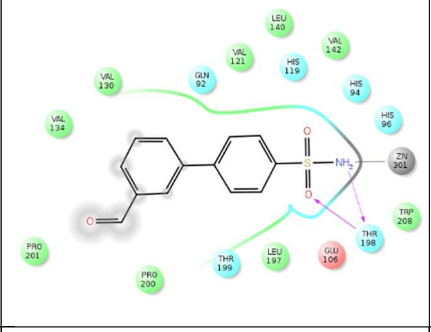	
5sz3	5sz6	

Table S2. Structures of the 70 ligands used as a first external test set.

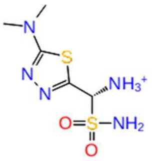	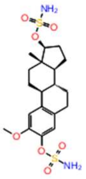	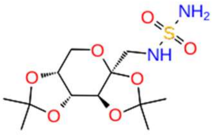
**2eu2**	**2gd8**	**2h15**
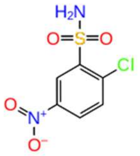	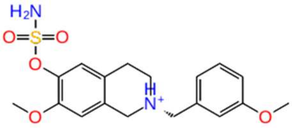	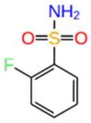
**2qp6**	**2wd2**	**2weg**
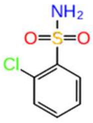	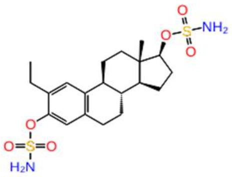	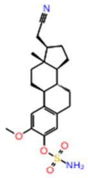
**2weh**	**2x7t**	**3bet**
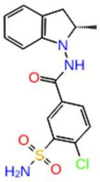	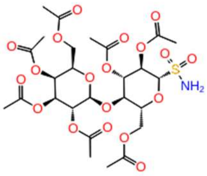	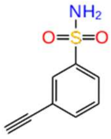
**3bl1**	**3hkn**	**3kig**
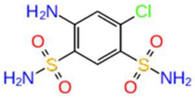	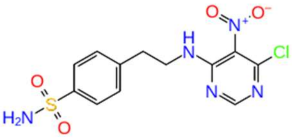	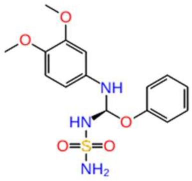
**3l14**	**3m2n**	**3m2x**
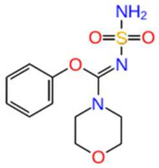	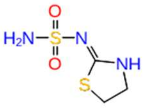	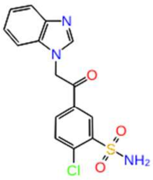
**3m04**	**3m14**	**3m98**
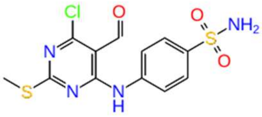	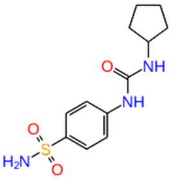	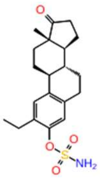
**3mho**	**3mzc**	**3oku**

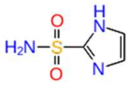	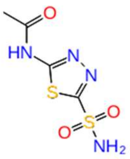	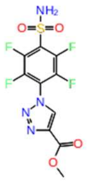
**3s76**	**3v2m**	**4dz7**
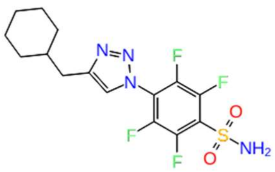	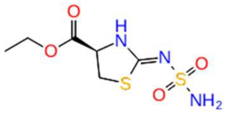	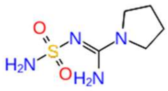
**4dz9**	**4fpt**	**4frc**
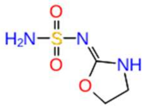	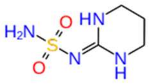	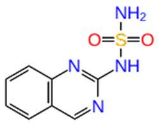
**4fu5**	**4fvn**	**4fvo**
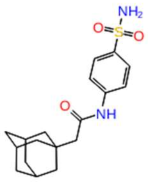	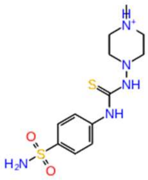	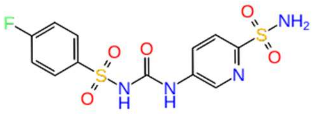
**4ilx**	**4ito**	**4kuw**
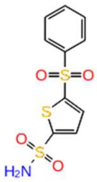	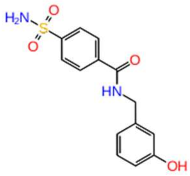	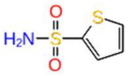
**4lhi**	**4mty**	**4n0x**
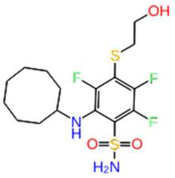	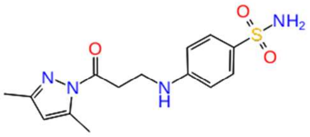	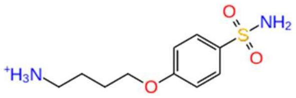
**4pyx**	**4q6e**	**4rfd**
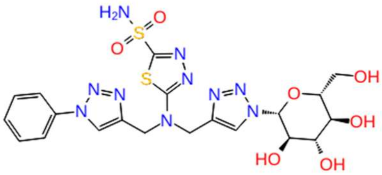	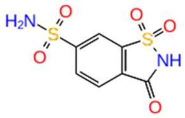	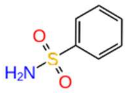
**4rn4**	**4xe1**	**4yx4**

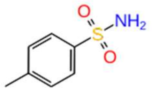	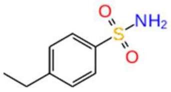	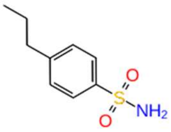
**4yxi**	**4yxo**	**4yxu**
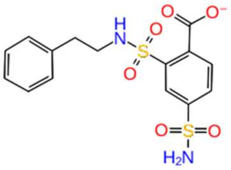	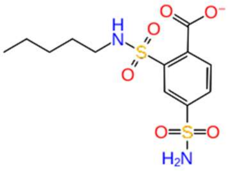	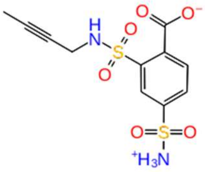
**5amd**	**5amg**	**5aml**
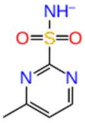	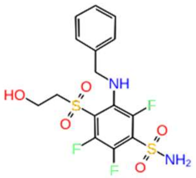	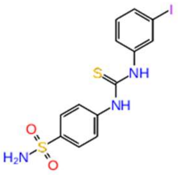
**5eh5**	**5ehe**	**5eij**
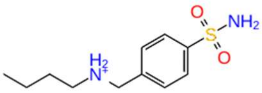	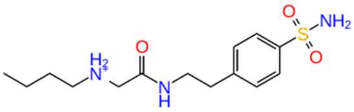	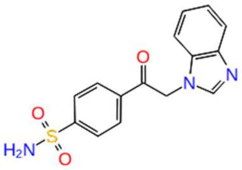
**5ekh**	**5ekj**	**5ll4**
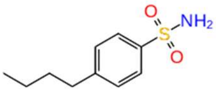	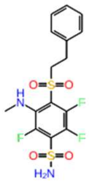	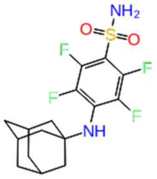
**5ll8**	**5llc**	**5lle**
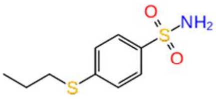	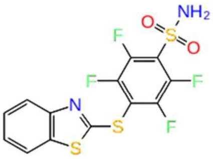	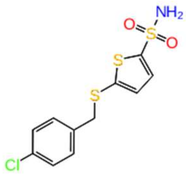
**5llg**	**5llh**	**5mjn**
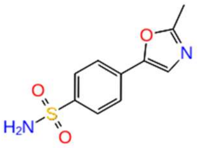	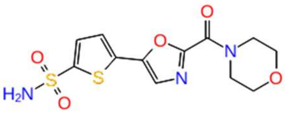	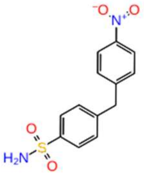
**5nea**	**5nee**	**5nx0**

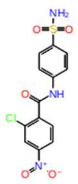	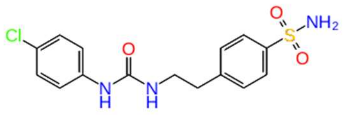	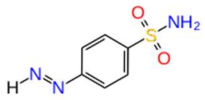
**5nxg**	**5ny3**	**5t71**
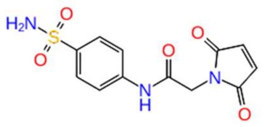	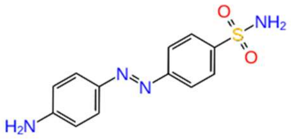	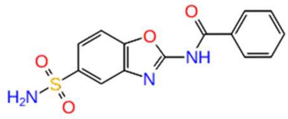
**5t74**	**5t75**	**5tfx**
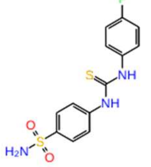		
**5uln**		

Table S3. Structures of the 15 ligands used as additional external test set.

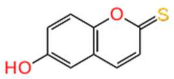	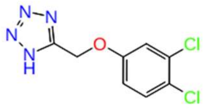	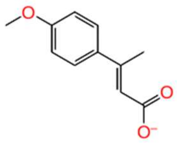
**4wl4**	**5eh7**	**5eh8**
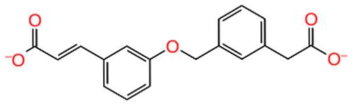	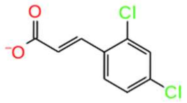	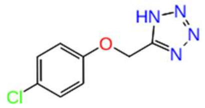
**5ehv**	**5ehw**	**5flo**
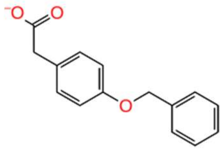	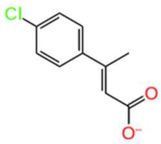	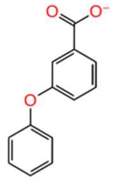
**5flq**	**5fls**	**5flt**
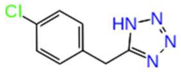	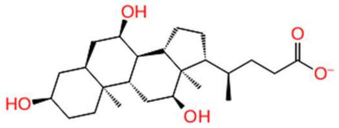	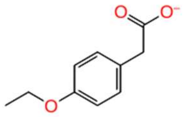
**5fng**	**4pxx**	**5fnj**
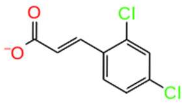	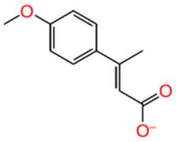	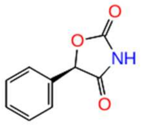
**5fnk**	**5fnm**	**5txy**
